# Nicotinamide Mononucleotide: A Promising Molecule for Therapy of Diverse Diseases by Targeting NAD+ Metabolism

**DOI:** 10.3389/fcell.2020.00246

**Published:** 2020-04-28

**Authors:** Weiqi Hong, Fei Mo, Ziqi Zhang, Mengyuan Huang, Xiawei Wei

**Affiliations:** ^1^Laboratory of Aging Research and Cancer Drug Target, State Key Laboratory of Biotherapy, National Clinical Research Center for Geriatrics, West China Hospital, Sichuan University, Chengdu, China; ^2^West China Hospital and State Key Laboratory of Biotherapy, Sichuan University, Department of Biotherapy, Chengdu, China

**Keywords:** nicotinamide adenine dinucleotide (NAD), nicotinamide mononucleotide (NMN), aging, diabetes, obesity, Alzheimer’s disease

## Abstract

NAD+, a co-enzyme involved in a great deal of biochemical reactions, has been found to be a network node of diverse biological processes. In mammalian cells, NAD+ is synthetized, predominantly through NMN, to replenish the consumption by NADase participating in physiologic processes including DNA repair, metabolism, and cell death. Correspondingly, aberrant NAD+ metabolism is observed in many diseases. In this review, we discuss how the homeostasis of NAD+ is maintained in healthy condition and provide several age-related pathological examples related with NAD+ unbalance. The sirtuins family, whose functions are NAD-dependent, is also reviewed. Administration of NMN surprisingly demonstrated amelioration of the pathological conditions in some age-related disease mouse models. Further clinical trials have been launched to investigate the safety and benefits of NMN. The NAD+ production and consumption pathways including NMN are essential for more precise understanding and therapy of age-related pathological processes such as diabetes, ischemia–reperfusion injury, heart failure, Alzheimer’s disease, and retinal degeneration.

## Introduction

Nicotinamide adenine dinucleotide (NAD) is a vital metabolic redox co-enzyme found in eukaryotic cells and is necessary for over 500 enzymatic reactions. It plays a crucial role in various biological processes, including metabolism, aging, cell death, DNA repair, and gene expression (Rajman et al., 2018; Okabe et al., 2019). Thus, NAD+ is critical for human health and longevity.

The co-enzyme was first discovered by Harden and Young in 1906 as a component that enhanced the rate of alcohol fermentation in yeast extracts (Harden and Young, 1906). Over subsequent years, the chemical composition of the co-enzyme was established as an adenine, a reducing sugar group and a phosphate by Hans von Euler-Chelpin (von Euler and Myrback, 1930). Then, in 1936, Warburg suggested that NAD+ could play a role in redox reactions (Warburg and Christian, 1936). By 1960, it was assumed that all biochemical investigations on NAD+ had been exhausted. In 1963, Chambon and Mandel reported that NAD+ is a co-substrate for the addition of poly-ADP-ribose to proteins, and this prompted a series of studies on poly-ADP ribose and poly-ADP-ribose polymerases (PARPs) (Chambon et al., 1963; Yoshino et al., 2018). In the last decade, new interests in NAD+ emerged because of its association with sirtuins, a family of NAD-dependent protein deacylases (SIRT1–7) (Rajman et al., 2018). Roy Frye showed that mammalian sirtuins could metabolize NAD+ and that NAD+ had a protein ADP-ribosyltransferase activity (Frye, 1999). Guarente and Imai made a phenomenal discovery that yeast SIR2 (silent information regulator 2) and the mouse ortholog SIRT1 have NAD+-dependent protein deacetylase activity (Imai et al., 2000). Previously, several studies had shown that sirtuins play a critical role in regulating multiple cellular functions, such as cell growth, energy metabolism, stress resistance, inflammation, and circadian rhythm neuronal function, among others (Imai and Yoshino, 2013; Rajman et al., 2018). The deficiency of NAD+ is closely associated with diverse pathophysiologies, including type 2 diabetes (T2D), obesity, heart failure, Alzheimer’s disease (AD), and cerebral ischemia. The NAD+ levels decline in multiple organs with age, and this contributes to the development of various age-related diseases (Yoshino et al., 2011; Gomes et al., 2013; Mouchiroud et al., 2013; Mills et al., 2016). Therefore, NAD+ supplementation could be an effective therapy for the treatment of the conditions mentioned above.

Nicotinamide mononucleotide (NMN) is one of the intermediates in NAD+ biosynthesis and is a bioactive nucleotide formed by the reaction between a phosphate group and a nucleoside containing ribose and nicotinamide (NAM) (Poddar et al., 2019). NAM is directly converted to NMN by nicotinamide phosphoribosyltransferase (NAMPT). The molecular weight of NMN is 334.221 g/mol (Poddar et al., 2019). There are two anomeric forms of NMN named alpha and beta, and the latter is the active form (Poddar et al., 2019). NMN is found in various types of natural foods, such as vegetables, fruits, and meat. Edamame and broccoli contain 0.47–1.88 and 0.25–1.12 mg NMN/100 g, respectively, whereas avocado and tomato contain 0.36–1.60 and 0.26–0.30 mg NMN/100 g, respectively. However, raw beef only contains 0.06–0.42 mg NMN/100 g (Mills et al., 2016). Recent preclinical studies have demonstrated that the administration of NMN could compensate for the deficiency of NAD+, and NMN supplementation was able to effect diverse pharmacological activities in various diseases.

In this review, NAD+ biosynthesis pathways and the possible reason for its age-related decline are described. Also, a summary of studies on the role of NAD+ deprivation in causing human diseases and how the application of NMN could have positive effects on those diseases is provided.

## NAD+ Biosynthesis Pathways

**FIGURE 1:**
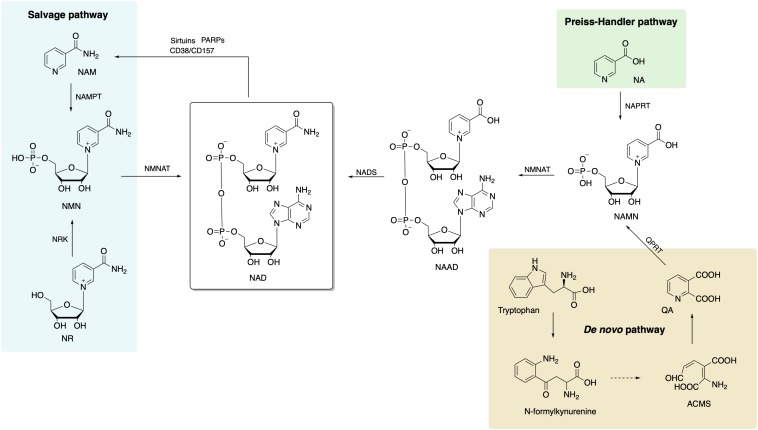
Biosynthetic pathways of NAD+ in mammalian cells includes *de novo*, Preiss–Handler, and salvage pathways, and the salvage pathway is the main source of NAD+. NAD, nicotinamide adenine dinucleotide; NA, nicotinic acid; NAPRT, nicotinic acid phosphoribosyltransferase; NAMN, nicotinic acid mononucleotide; NAAD, nicotinic acid adenine dinucleotide; NADS, NAD+ synthetase; NMNAT, nicotinamide/nicotinic acid mononucleotide adenylyltransferase; ACMS, 2-amino-3-carboxymuconate semialdehyde; QA, quinolinic acid; QPRT, quinolinate phosphoribosyltransferase; NAM, nicotinamide; NAMPT, nicotinamide phophoribosyltransferase; NMN, nicotinamide mononucleotide; NR, nicotinamide riboside; NRK, nicotinamide riboside kinase.

Three different NAD+ biosynthesis pathways have been described in mammalian cells (Figure 1): (1) Preiss–Handler, in which NAD is synthesized from nicotinic acid (NA); (2) *de novo* synthesis, which starts from tryptophan; and (3) salvage pathway, which is most predominant in mammalian cells.

### The Preiss–Handler Pathway

This pathway starts with conversion of NA to the nicotinic acid mononucleotide (NAMN) by the enzyme nicotinic acid phosphoribosyltransferase (NAPRT) (Preiss and Handler, 1958). Afterward, NAMN is used for nicotinic acid adenine dinucleotide (NAAD+) biosynthesis by nicotinamide/nicotinic acid mononucleotide adenylyltransferase (NMNAT1/2/3). Finally, NAD+ synthetase (NADS) transforms NAAD+ to NAD+ with ammonia and ATP action as extra ingredients (Yang and Sauve, 2016).

### *De novo* Synthesis From Tryptophan

The eight-step *de novo* synthesis pathway is initiated by indoleamine 2,3-dioxygenase (IDO) or tryptophan 2,3-dioxygenase (TDO) that convert tryptophan to *N*-formylkynurenine (Salter et al., 1991). Through formamidase (KFase), *N*-formylkynurenine is then transformed to be kynurenine, to which hydroxyl is added via kynurenine 3-hydroxylase (K3H). The product, 3-hydroxy-kynurenine, is converted to 3-hydroxyanthranilate followed by 2-amino-3-carboxymuconate semialdehyde (ACMS) via kynureninase (Kyase) and 3-hydroxyanthranilate-3,4-dioxygenase. ACMS then cyclizes to form quinolinic acid (QA) that participates in NAMN biosynthesis with quinolinate phosphoribosyltransferase (QPRT) (Yang and Sauve, 2016). The last two steps are the same as the Preiss–Handler pathway that NAD+ is synthetized sequentially from NAMN and NAAD by NMNAT 1/2/3 and NADS.

### The Salvage Pathway

The salvage pathway is the primary source of NAD+ in mammalian cells. The degradation of NAD+ and subsequent generation of NAM (as a by-product) are achieved by NAD-consuming enzymes, such as sirtuins, PARPs, CD38, CD157, and sterile alpha and TIR motif-containing protein 1 (SARM1) (Okabe et al., 2019). There are only two steps in the salvage pathway. The rate of NAD+ synthesis in this pathway is mostly determined by NAMPT that converts NAM and 5-phosphoribosyl-1-pyrophosphate (PRPP) to NMN in the first step. Then, NMN, the substrate for NAMNT, is conjugated to ATP and converted to NAD in the second step.

The NAMPT exists in two forms in mammals, that is, intracellular NAMPT (iNAMPT) in the cytoplasm and nucleus and extracellular NAMPT (eNAMPT) in the plasma or extracellular space (Revollo et al., 2007). The SIRT1-dependent deacetylation of iNAMPT predisposes the protein to secretion in adipocytes (Yoon et al., 2015). Various types of cells, including mature adipocytes, pancreatic β-cells, myocytes, epithelial cells, and hepatocytes (Revollo et al., 2007; Garten et al., 2010; Zhao et al., 2014) secrete and release eNAMPT to the plasma or extracellular space.

Also, another NAD precursor, nicotinamide riboside (NR), is incorporated into cells using equilibrative nucleoside transporters (ENTs) (Nikiforov et al., 2011) and phosphorylated to NMN by nicotinamide riboside kinase (NRK1/2) intracellularly (Ratajczak et al., 2016). Conversion of extracellular NMN to NR mediated by enzyme CD73 is required for cell uptake and intracellular synthesis of NAD+ (Grozio et al., 2013; Ratajczak et al., 2016). NAD+ biosynthesis in kidney and brown adipose tissue has been shown to decrease after administration of NMN in NRK1 knockout mice (Ratajczak et al., 2016). However, a recent study identified Slc12a8 as a specific transporter of NMN, which is highly expressed in the small intestine (Grozio et al., 2019). In the study, Slc12a8 expression was upregulated in the small intestines of aged mouse in response to a decrease of NAD+. These findings suggested that the uptake pathway of NMN could be via a cell- or tissue-specific manner.

Given that the salvage pathway is the main and the most efficient route for NAD+ biosynthesis, NMN or NR supplementation is becoming the preferred option of improving NAD+ levels that is devoid of side effects. Currently, increasing numbers of clinical trials using NMN and NR have been approved and are geared toward the treatment of various diseases, which further demonstrate that NMN is a suitable and safe drug for use in humans.

## Effect of Aging on NAD+ Levels

### NAD+ Biosynthetic Pathways Decline With Age

The decline in NAD+ biosynthetic pathways in the course of aging could be a possible explanation for the reduction of NAD+ levels. NAMPT controls NAD+ levels, thereby influencing the activity of NAD-dependent enzymes, including sirtuins and PARPs.

**FIGURE 2:**
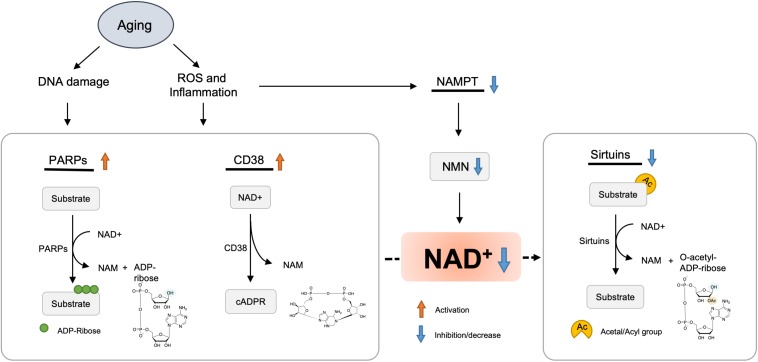
Hypothetic molecule mechanisms of NAD+ decreased with aging. Oxidative stress, DNA damage, and chronic inflammation are increased with aging, which results in accelerated NAD degradation via activation of CD38 and PARPs, or dysregulation of NAMPT. Finally, decreased levels of NAD+ lead to various metabolic and age-associated diseases.

A study demonstrated that the NAD+ levels and NAMPT protein levels declined significantly in multiple organs, including the pancreas, white adipose tissue (WAT), and skeletal muscle of old mice (Yoshino et al., 2011). However, exercise training increased NAMPT expression in the skeletal muscles (Costford et al., 2010). NAD+ levels and exercise capacity were preserved in aged transgenic mice with muscle-specific NAMPT transgene expression (Frederick et al., 2016). These results suggested that the deficiency of NAMPT result in a reduction of NAD+ levels in the aged mice, and exercise may elevate NAMPT expression, thus restoring the NAD+ levels (Figure 2).

Inflammation and oxidative stress caused by aging have been shown to reduce the NAMPT-mediated NAD+ biosynthesis (Yoshino et al., 2011; Figure 2). Besides, *Nampt* gene encoding is controlled by BMAL1/CLOCK complex, a heterodimeric complex of core circadian transcription factors, which is suppressed by inflammatory cytokines (Cavadini et al., 2007). Therefore, the development of chronic inflammation in the course of aging may contribute to the inhibition of NAMPT-mediated NAD+ biosynthesis and CLOCK/BMAL-mediated circadian machinery (Imai and Guarente, 2014).

### NAD+-Consuming Enzymes Are Activated With Age

#### PARP1

PARPs were initially considered to be DNA damage repair agents in the 1960s (Chini et al., 2017). The accumulation of DNA damage during aging could activate PARP, among which PARP-1 acts as a major cellular NAD+-consuming enzyme (Imai and Guarente, 2014). Cockayne syndrome (CS) is an aging-related progressive neurodegeneration that occurs as a result of mutations in either Cockayne syndrome group A (CSA) or B (CSB) proteins (Gitiaux et al., 2015; Scheibye-Knudsen et al., 2014). In CS mice, PARP inhibitor or NAD+ supplementation reversed decline in SIRT1 activation and mitochondrial function caused by aberrant PARP activation (Scheibye-Knudsen et al., 2014). Consistently, another inhibitor of PARP, PJ34, or knockout boosted the levels of NAD+, SIRT1 activity, and oxidative metabolism (Bai et al., 2011).

#### CD38

The CD38 enzyme and its homolog CD157 were initially described as plasma membrane antigens on thymocytes and T lymphocytes. Their role in NAD+ consumption have been revealed; that is, CD157/BST-1 could hydrolyze NR (Preugschat et al., 2014) and CD38 hydrolyzes NAD+ to generate NAM, adenosine diphosphoribose (ADPR), and cyclic ADPR (cADPR). In addition, CD38 also hydrolyzes cADPR (De Flora et al., 2004) and NMN (Grozio et al., 2013).

In mammals, the level of NAD+ and mitochondrial function decreased partially through regulation of SIRT3 as the expression and activity of CD38 protein increased in various tissues during aging (Camacho-Pereira et al., 2016). Administration of CD38 inhibitors elevated intracellular NAD+ level (Escande et al., 2013; Boslett et al., 2017). Consistently, CD38 knockout mice displayed significantly higher NAD+ level in multiple organs (Young et al., 2006).

#### Sterile Alpha and TIR Motif-Containing 1 (SARM1) Protein

The toll/interleukin-1 receptor (TIR) domain of sterile alpha and TIR motif-containing 1 (SARM1) protein presents NADase activity (Rajman et al., 2018) that is involved in axonal degeneration after axon injury. In response to neuronal injury, the TIR domain of SARM1 cleaves NAD+ to generate ADP ribose (ADPR) and cyclic ADPR, which may contribute to axonal degeneration (Essuman et al., 2017). Paradoxically, overexpression of enzymes in NAD+ biosynthesis pathway or supplying NR could inhibit SARM1-induced axon destruction (Gerdts et al., 2015).

**FIGURE 3:**
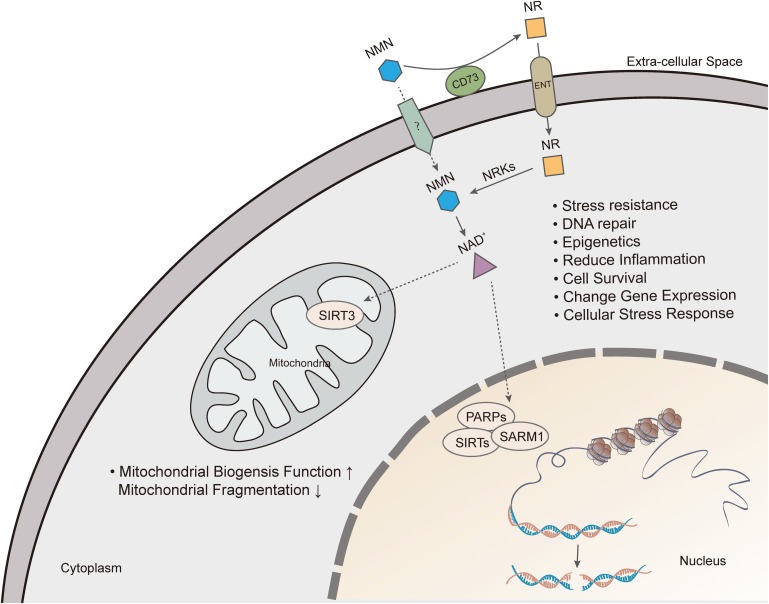
Nicotinamide mononucleotide exerts pharmacological effects by increasing intracellular NAD+ levels. Extracellular NMN is cleavage by CD73, which yields NR that is incorporated into cells using equilibrative nucleoside transporters (ENTs). NMN is converted to NAD+, which produces beneficial effects on cell, including mitochondrial function, DNA repair, gene expression, anti-inflammation and cell survival.

In summary, there are many ways of restoring NAD+ level depletion caused by aging or other diseases, including improving NAMPT expression, providing NAD+ precursors, or inhibiting NAD+, consuming enzymatic activities of PARP, CD38, and SARM1. Currently, supplementation with NMN or NR is considered a viable and highly efficient strategy of increasing NAD+ levels (Figure 3).

## Diabetes

The global prevalence of diabetes has increased dramatically over the past four decades. According to the WHO report, the number of people with diabetes rose from 108 million in 1980 to 422 million in 2014. T2D is characterized by insulin resistance and subsequent impairment of insulin secretion (Okabe et al., 2019). The metabolism of NAD+ plays a crucial role in insulin sensitivity and secretion and is sometimes disrupted by obesity and aging.

Revollo et al. showed that eNAMPT was necessary for NAD+ biosynthesis (Revollo et al., 2007). Declined NAD+ levels and glucose-stimulated insulin secretion (GSIS) in pancreatic β cells and impaired glucose tolerance were observed in Nampt^+/–^ mice. Similar blood glucose and plasma insulin levels were observed in Nampt^+/–^ and control mice after NMN (i.p. 500 mg/kg) treatment. Also, FK866, an inhibitor of NAMPT, reduced NAD+ levels, and glucose-stimulated insulin secretion in primary islets, whereas NMN treatment reversed the defects (Revollo et al., 2007). These results demonstrated that Nampt-mediated NAD+ biosynthesis is critical for β cell function, and that NMN treatment can ameliorate the deficiency in NAD biosynthesis and glucose-stimulated insulin secretion.

Obesity and diabetes are inextricably linked. MicroRNAs (miRNAs) are key regulators of metabolism, by which SIRT1 expression is regulated in healthy conditions and metabolic diseases (Lee and Kemper, 2010). In the dietary obese mice, the elevation of hepatic microRNA-34a (miR-34a) inhibited the expression of NAMPT and SIRT1, which was responsible for the decrease in NAD+ levels and SIRT1 activity (Choi et al., 2013). The reduction of SIRT1 activity resulted in transcriptional responses of decreased fatty acid β-oxidation and increased lipogenesis and inflammation (Choi et al., 2013). Mice overexpressing miR-34a were intraperitoneally injected with NMN (500 mg/kg) for 10 days consecutively, the effects caused by hepatic overexpression of miR-34 were reversed, and glucose tolerance was enhanced (Choi et al., 2013). These results suggested that NMN could be a potential agent for the treatment of obesity-associated T2D involving SIRT1 dysfunction.

Because of the high intake of dietary sugar, the risk of metabolic syndrome and T2D has been on the increase in humans (Malik et al., 2010). Fructose consumption contributes to the development of pro-inflammatory effect in rodent models, which is involved in the process of insulin resistance and the onset of T2D (Roncal-Jimenez et al., 2011). Fructose-rich diet (FRD) results in T2D-like symptoms, including hyperglycemia, dyslipidemia, and inflammation (Roncal-Jimenez et al., 2011). FRD-fed mice showed an increased expression of IL-1b and TNF-α, which are pro-inflammatory phenotypes. The GSIS and leucine-stimulated insulin secretion (LSIS) were significantly reduced in FRD-fed mice, which was associated with islet dysfunction caused by a decrease of eNAMPT in plasma, whereas the administration of NMN at the dose of 500 mg/kg eliminated the adverse effects of FRD on GSIS and LSIS in mice (Caton et al., 2011).

FRD increased the expression of Inos (induces cellular stress and cell death) and Bax (pro-apoptotic gene) genes and reduced the expression of Pdx1, Glut2, and Gk genes, which are all essential for glucose detection and beta-cell differentiation. These changes in gene expression were restored by NMN treatment. Moreover, the decrease in expression of Sirt1 and Sirt3 genes in FDR mice were reversed by NMN treatment. These results suggested that NMN could improve islet function by influencing the expression of genes related to anti-inflammatory, islet beta-cell differentiation, and SIRT1 activation.

Yoshino et al. revealed that the administration of NMN was highly effective in combating diet- and age-induced T2D (Yoshino et al., 2011). In their study, mice fed with high-fat diet (HFD) displayed significantly reduced NAMPT protein and NAD+ levels in the liver and WAT and not in skeletal muscle as was expected. The HFD-induced male and female diabetic mice after receiving intraperitoneal administration of NMN (500 mg/kg/day) for 10 and 7 consecutive days, respectively, exhibited restored NAD+ levels in the liver and WAT. Impaired glucose and insulin tolerance were significantly improved in diabetic female mice. The effect of NMN in reversing impaired glucose tolerance was milder in males compared to females, and insulin tolerance remained unchanged in male mice.

Yoshino et al. also confirmed that NMN improves hepatic insulin sensitivity by reversing the expression of genes related to oxidative stress, inflammatory response, immune response, and lipid metabolism (Yoshino et al., 2011). For example, the expression of the glutathione *S*-transferase alpha two gene (Gsta2), which is crucial for the maintenance of hepatic insulin by protecting lipid peroxidation products, was suppressed by HFD but activated by NMN. Other genes that are related to insulin resistance were significantly influenced by NMN in HFD-induced mice, such as interleukin 1β, lipin1, and pyruvate dehydrogenase kinase 4 (Pdk4). Also, Yoshino et al. revealed that SIRT1 was responsible for gene expression dynamics, and its suppression by HFD was restored by NMN.

Aging is one of the highest risk factors for developing T2D (Moller et al., 2003). Previous studies have shown that progressive decline in β cell function in the course of aging contributes to the pathophysiology of T2D (Basu et al., 2003). As mentioned above, NAD+ and NAMPT levels declined during aging (Yoshino et al., 2011). Administration of NMN (500 mg/kg/day) for 11 consecutive days resulted in significant improvement in glucose tolerance and utilization in aged mice. Also, hyperlipidemia induced by HFD was also reversed by this treatment. Meanwhile, NMN did not have other effects on glucose homeostasis in non-diabetic old mice (Yoshino et al., 2011). Mills et al. (2016) also demonstrated that long-term (12 months) administration of NMN ameliorated age-associated decreased insulin sensitivity. Triglyceride levels in the liver were lower after 12-month NMN administration, which indicated a decline in insulin resistance. Mice that were put on long-term NMN intervention showed a tendency of plasma fatty acids (FFA), which is consistent with improved insulin sensitivity (Mills et al., 2016).

Moynihan et al. (2005) reported that an increase in Sirt1 dosage in pancreatic β cells improved GSIS and glucose tolerance in beta cell-specific Sirt1-overexpression (BESTO) transgenic mice at 3 and 8 months of age. However, the same cohort of BESTO mice did not show these beneficial effects at 18–24 months of age (Ramsey et al., 2008). It has been reported that Sirt1 improves glucose-stimulated insulin secretion through the repression of Ucp2 and improvement of ATP levels in pancreatic β cells (Bordone et al., 2006; Ramsey et al., 2008), which was abolished in aged mice, resulting in maintenance of a high level of Sirt1 protein. Also, NMN plasma levels declined significantly in aged BESTO mice, which suggested that a decrease in Sirt1 activity and loss of the glucose-responsiveness in BESTO mice resulted from the decline in systemic NAD biosynthesis. Thus, intraperitoneal injection of NMN (500 mg/kg) into 20-month-old BESTO mice led to an enhancement in GSIS and improvement in glucose tolerance in the aged BESTO females instead of males.

Generally, these discoveries demonstrated that NMN could be a promising drug for obese-associated and age-induced T2D through the role it plays in the enhancement of NAD+ biosynthesis and Sirt1 activity.

## Obesity

Obesity is associated with insulin resistance in multiple organs. It is a systemic metabolic derangement, which is involved in the pathogenesis of many diseases such as T2D, non-alcoholic fatty liver disease (NAFLD), atherogenic dyslipidemia, and cardiovascular disease (Reaven, 1988). It has been reported that the dysfunction of adipose tissue could result in obesity-associated metabolic disorders in multiple organs because adipose tissue has effects on maintaining the functional integrity of whole-body metabolic health (Stromsdorfer et al., 2016). Significantly reduced iNAMPT has been manifested in adipocyte as a result of HFD (Yoshino et al., 2011; Chalkiadaki and Guarente, 2012), however, elevated eNAMPT was detected in obesity (Catalán et al., 2011). Compared with clear function of iNAMPT in NMN synthesis, the significance of eNAMPT is controversial. In response to cellular stress, nutritional cues, or inflammatory cytokines, adipocytes secreted eNAMPT through PI3K-AKT pathway (Haider et al., 2006), SIRT1-mediated pathway (Yoon et al., 2015), or other unknown pathway (Tanaka et al., 2007). The role of eNAMPT has been indicated to be inflammatory promotion, inflammatory suppression (Revollo et al., 2007; Li et al., 2008; Pillai et al., 2013; Zhao et al., 2013; Jing et al., 2014), enhancement of food intake (Brunetti et al., 2012), and regulation of insulin resistance as well as plasma free fatty acid concentration (Stromsdorfer et al., 2016; Nielsen et al., 2018). The function of eNAMPT remains elusive in recent research, demonstrating that low concentration of dimeric eNAMPT benefited for beta cell function through NAD+, however, a higher monomeric eNAMPT level presented hostile effect on beta cell function (Sayers et al., 2020). Obesity has also been associated with the dampened NAD+/SIRT pathway in adipose tissue (Jukarainen et al., 2016). These findings suggested that NAMPT-mediated NAD+ biosynthesis in adipose tissue could be involved in the regulation of whole-body glucose metabolism.

Adipocyte-specific Nampt knockout (ANKO) mice showed a severe multi-organ insulin resistance, including adipose tissue, liver, and skeletal muscle, independently with an increase in whole-body adiposity and weight. The plasma FFA availability and local adipose tissue inflammation were also increased in the ANKO mice (Stromsdorfer et al., 2016). The plasma concentration of two key adipokines, namely, adiponectin and adipsin, were significantly reduced in ANKO mice. It has been reported that adiponectin and adipsin regulate insulin sensitivity and glucose homeostasis (Kadowaki et al., 2006). Phosphorylation of cyclin-dependent kinase 5 (CDK5) and peroxisome proliferator-activated receptor γ (PPARγ) was increased in adipose tissue of the ANKO mice, which led to a significant decline in gene expression of obesity-linked specific targets of phosphorylated PPARγ, including adiponectin and adipsin. As was expected, NAD+ levels in adipose tissue from the ANKO mice were significantly increased after oral administration of NMN (500 mg/kg) for 4–6 weeks. The NMN treatment also improved multi-organ insulin sensitivity and normalized plasma insulin and FFA concentrations in the ANKO mice. Moreover, the phosphorylation of PPARγ (Ser273) and CDK5 in visceral adipose tissue (VAT) was reduced by the NMN treatment. Accordingly, the plasma concentrations and gene expression of adiponectin and adipsin in adipose tissue were enhanced (Stromsdorfer et al., 2016). Therefore, these results provided evidence that NMN could be a therapeutic molecule for obesity-associated systemic metabolic derangements, particularly multi-organ insulin resistance. ANKO and brown adipocyte-specific Nampt knockout (BANKO) mice both showed impaired gene programs involved in thermogenesis, mitochondrial biogenesis, and FFA metabolism in BAT (Yamaguchi et al., 2019). However, only ANKO mice have a blunted thermogenic response (lower temperature in rectal and BAT, and whole-body oxygen consumption) to acute cold exposure, fasting, and administration of β-adrenergic agonists. Altered function in WAT may contribute to this difference. Lack of NAMPT in WAT decreased adrenergic-mediated lipolysis through inactivation of the NAD+–SIRT1–caveolin-1 axis, which reduced the release of FFAs as fuel source for BAT thermogenesis. NMN administration normalized these metabolic abnormalities, including increasing BAT NAD+ levels, decreasing BAT weight and BAT whitening as well as restoring gene expression of caveolin-1 in WAT and those involved in thermogenesis, mitochondrial function, and FFA metabolism in BAT. Moreover, ANKO mice treated with NMN showed greater cold tolerance compared with ANKO mice (Yamaguchi et al., 2019).

Several studies have confirmed that physical exercises have various health benefits, especially in obesity-related cases (Vieira et al., 2009; Ross et al., 2015), which, in part, results from the upregulation of mitochondrial activity because of increased NAD levels. Mouse models showed that NAD+ levels in metabolic organs and mitochondrial biogenesis could be improved by physical exercises (Ross et al., 2015). Thus, taking physical exercises is thought to be an effective way of increasing NAD+ levels. To compare the efficiency of increasing NAD+ levels between NMN supplementation and exercise, HFD-induced obese mice were given NMN (500 mg/kg) for 17 days or treadmill running (45 min/day) was implemented for 6 days per week for 6 weeks. According to the results, NMN treatment increased NAD+ levels in muscles and the liver, but exercise increased NAD+ levels in muscles only. The liver mass and triglyceride content were significantly reduced, and citrate synthase activity was increased after NMN treatment in HFD-fed mice, which suggested that NMN could increase catabolism of fats. Exercises and NMN have a similar effect on glucose intolerance induced by obesity, however, these two interventions are tissue-specific with a different impact on mitochondrial function in muscles and the liver (Uddin et al., 2016). Uddin et al. (2017) also showed that NMN could reduce the effects of maternal obesity as compared to exercise. Maternal overnutrition is often associated with increased infant birth weight, adiposity, and high risk of long-term obesity in later life of the offspring (Castillo-Laura et al., 2015). Thus, it is important to investigate new strategies for reducing the risk in future generations. A study found that treadmill exercise (for 9 weeks) and NMN injection (for 15 days) both reduced adiposity and improved glucose tolerance and mitochondrial function. Moreover, NMN showed stronger effects on liver fat catabolism and synthesis than exercise. This study suggested that NMN treatment might be an effective option for reversing negative effects caused by maternal obesity (Uddin et al., 2017).

Long-term administration of NMN can significantly reduce age-associated body weight gain in a dose-dependent manner. A study conducted a 12-month-long administration of NMN (from 5 to 17 months) in mice. The results indicated that the 100- and 300-mg/kg dose of NMN was able to reduce the mice’s weight by 4 and 9%, respectively, compared to the control mice. No difference was observed in body length between NMN-treated and control mice. NMN-treated mice also maintained higher levels of food and water consumption compared to control mice, which suggested that NMN did not cause severe side effects, such as growth defect and loss of appetite (Mills et al., 2016). In conclusion, the administration of NMN could be an effective option for maintaining body weight and reversing metabolic dysfunctions caused by obesity.

## Ischemia–Reperfusion Injury

Ischemia is known to decrease oxygen and ATP levels in tissues, leading to cell necrosis. Reperfusion is a reoxygenation process whereby blood re-enters a previously ischemic tissue, and this often causes calcium overload and production of ROS (Sanada et al., 2011). Ischemia followed by reperfusion has been reported to trigger severe tissue damage, for which ischemic preconditioning (IPC) is a confirmed preventative strategy (Sanada et al., 2011).

It has been reported that activation of SIRT1 can protect the heart from ischemia and reperfusion (I/R)-induced injury (Hsu et al., 2010). SIRT1 upregulates cardioprotective molecules, such as MnSOD (antioxidants), Trx1 (antioxidants), and Bcl-xL (anti-apoptotic), while it decreases pro-apoptotic molecules, including Bax and cleaved caspase-3. FoxO1, a transcription factor deacetylated by Sirt1, partially mediates the SIRT1-induced upregulation of MnSOD (Hsu et al., 2010), which protects the heart from oxidative stress. Moreover, maintaining NAMPT expression is critical for prevention of myocardial injury caused by I/R (Hsu et al., 2009). The deacetylase activity of SIRT1 is dependent on NAD+. Thus, enhancing NAD+ may promote SIRT1-mediated IPC.

NMN has been shown to confer protection on the heart in ischemic and reperfusion conditions (Yamamoto et al., 2014). Yamamoto et al. found that IPC upregulates NAMPT and the protective effect of IPC on heart during I/R injury is attenuated in Nampt^+/–^ mice, suggesting that NAMPT mediates the protective effect of IPC. The NAD+ levels in heart were decreased after 30 min of ischemia and transiently normalized by NMN administration. Implementation of two patterns of NMN administration, once 30 min before ischemia or 4 times repetitive administration just before and during reperfusion, reduced the infarct size by 44 and 29%, respectively. However, a single administration of NMN 12 h before ischemia and once immediately before reperfusion did not significantly reduce the infarct size. These findings suggest that NMN reduces infarct size after by I/R, and this effect is timing-dependent. Notably, NMN attenuated the increase in acetylation of FoxO1 during myocardial ischemia, but NMN failed to reduce the infarct size in Sirt1-KO mice, indicating that the protective effect of NMN on the heart following I/R injury is partly mediated by Sirt1.

NMN suppressed cardiomyocyte apoptosis in the peri-infarct area after I/R and significantly improves left ventricle (LV) systolic function caused by I/R. In addition, NMN activates autophagy during myocardial ischemia, which is consistent with the finding that NAMPT and SIRT1 promote autophagy in cardiomyocytes (Hsu et al., 2009). An *ex vivo* experiment showed that NMN attenuated myocardial I/R injury in aged rats (Hosseini et al., 2019b). Rats treated with NMN showed improved myocardial function and reduced infarct size. Besides, NMN exerted positive effects on mitochondrial function by improving the antioxidant system, restoring oxidative stress, and reducing mitochondrial ROS production and membrane depolarization. Importantly, the combination of NMN and melatonin could produce stronger cardioprotection.

Glycolytic stimulation during ischemia and enhanced acidosis during reperfusion are additional mechanisms for NMN-induced cardioprotection (Nadtochiy et al., 2018). Under normal conditions, cardiac ATP production is executed by β-oxidation of fatty acids (Stanley et al., 2005). Generating ATP via glycolysis is usually considered a manifestation of cardiac pathology and heart failure (Stanley et al., 2005). However, recent studies show that glycolysis has cardioprotective effects (Gohil et al., 2010). Nadtochiy et al. reported that NMN stimulates glycolysis and increases ATP production during ischemia, which partially contributes to NMN-induced cardioprotection (Nadtochiy et al., 2018). IR injury triggers the opening of the mitochondrial permeability transition pore (mPTP). Acid pH maintains the closed state of PT pores during ischemia, however, changes in pH during reperfusion promote mitochondrial pore opening (Griffiths and Halestrap, 1995). Addition of acidic media offers cardioprotection by maintaining the closed state of PT pore in early reperfusion (Cohen et al., 2007). It is worth noting that acidosis may promote mPTP in energetic mitochondria by stimulating Pi uptake (Kristian et al., 2001). NMN elevates cardiac lactate and pyruvate to induce acidosis, which protects against IR-induced injury (Nadtochiy et al., 2018).

Park et al. (2016) showed that NMN antagonizes global cerebral ischemia injury. Ischemic insults increase the production of free radicals, which causes DNA oxidative damage and PARP1 activation, and uncontrolled PARP1 activation decreases NAD+, which further decreases ATP synthesis, leading to cell death (Strosznajder et al., 2003). Conversely, NMN maintains normal cellular NAD+ levels by inhibiting the NAD+ catabolism of PARP1 to improve bioenergetics metabolism of post-ischemic tissue and ameliorate brain damage. NMN treatment at 30 min after initiation of reperfusion reduces hippocampal CA1 neuron cell death and improves ischemia-induced hippocampal dysfunction involved in spatial working memory (Park et al., 2016). The NAD+ levels in hippocampal tissue are significantly reduced after forebrain ischemia, and NMN inhibits this decrease in NAD+. Meanwhile, studies show that hippocampal PARP protein levels are significantly increased in mice with cerebral ischemia, accompanied by elevated ROS production. Interestingly, this effect is blocked by administration of NMN at the start of reperfusion. Notably, when administered at a dose of 62.5 mg/kg, NMN produced optimal treatment effects compared to other doses (500, 250, 125, and 31.25 mg/kg), suggesting that high dosage of NMN may introduce adverse effects on post-ischemic neurons. In fact, accumulation of NMN in nerve injury promotes axonal degeneration (Di Stefano et al., 2015) and NMN deamidase delays Wallerian degeneration and rescues axonal outgrowth defects (Di Stefano et al., 2017). Recently, Klimova et al. (2020) revealed a novel link between mitochondrial NAD+ metabolism, mitochondrial dynamics, and ROS production in cerebral ischemia. They showed that NMN treatment prevents post-ischemic depletion of mitochondrial NAD+, suppresses mitochondrial fragmentation, and reduces ROS generation via SIRT3-dependent mechanisms (Klimova et al., 2020). The activity of superoxide dismutase 2 (SOD2), a key mitochondrial antioxidant enzyme, was inhibited by an increase in its acetylation after ischemia, which can be reversed by NMN treatment. Moreover, NMN also prevents ischemia-induced phosphorylation of mitochondrial fission Dynamin-related protein (Drp1) (Klimova et al., 2020).

Wang P. et al. (2011) have already demonstrated that NAMPT protected against ischemic stroke through promoting neuronal survival via the SIRT1-dependent AMPK pathway. As the NAMPT enzymatic product, NMN alleviates cerebral infarction size, neurological deficit, and neuronal cell death (Wang P. et al., 2011). Furthermore, the important role of NAMPT–NAD cascade in regenerative neurogenesis after ischemic stroke has been underscored by Zhao et al. (2015), and delayed NMN supplementation for 7 days with the first administration at 12 h after cerebral ischemia improved post-ischemic regenerative neurogenesis.

## Heart Failure and Cardiomyopathies

Heart failure is the end stage of heart disease development and refers to the inability of the heart to pump sufficient blood to match the metabolic requirements of tissues due to impaired systole and/or diastole. Generally, heat failure is always associated with enlarged heart and dilated ventricles (Pillai et al., 2005).

Heart failure is one of diseases that are associated with mitochondrial respiratory dysfunction (Karamanlidis et al., 2013). It was found that deletion of Ndufs4, a protein critical for assembly and/or stability of complex I, resulted in a significant loss of complex I function in the heart. The cardiac-specific Ndufs4 KO (cKO) mice not only showed normal longevity and cardiac function in unstressed condition, but also exhibited normal myocardial energetics and contractile function during baseline workload or acute increases of workload. However, after chronic increases of workload including pressure overload and repeated pregnancy, the cKO mice with complex I deficiency developed heart failure and high cell death, and could not be explained by oxidative stress mechanism. Complex I deficiency significantly decreased NAD+/NADH ratio, which inhibited sirt3 activity, increased mitochondrial protein acetylation, and sensitized mPTP. Additionally, it has been shown that NMN treatment rescues NAD+/NADH ratio and mitochondria protein acetylation in cKO hearts, and normalizes the sensitivity of the mPTP (Karamanlidis et al., 2013).

Lee et al. reported that mitochondrial protein hyperacetylation, which is caused by elevated NADH/NAD+, increases the risk of development of heart failure by two distinct mechanisms (Lee et al., 2016). Malate aspartate shuttle (MAS) modulates communication between cytosolic and mitochondrial NAD+ redox states, transferring electrons from cytosolic NADH generated from glycolysis into mitochondria for oxidative phosphorylation (LaNoue and Williamson, 1971; Lee et al., 2016). During mitochondrial dysfunction, hyperacetylation of MAS decreases cytosolic NAD+/NADH ratio, thereby inducing mPTP-related cell death and the development of heart failure (Elrod et al., 2010). Lee et al. (2016) also identified that the acetylation of lysine-70 on oligomycin-sensitive conferring protein (OSCP) sensitized mPTP opening by promoting its interaction with cyclophilin D (CypD), a regulator of mPTP. Thus, administration of NMN reverses hyperacetylation of these proteins by normalizing the NAD+ redox balance, thereby protecting mice from heart failure.

Kruppel-like factor 4 (KLF4) is critical for cardiac mitochondrial homeostasis. Accordingly, mice with cardiac-specific deficiency of KLF4 (CM-K4KO) are more sensitive to pressure overload-induced heart failure (Liao et al., 2015). Zhang et al. found that cardiac KLF4 deficiency led to hyperacetylation of mitochondrial proteins, including SOD2, CypD, and long-chain Acyl-CoA dehydrogenase (LCAD), which impaired mitochondrial metabolic function and predisposed the CM-K4KO hearts to stress-induced dysfunction (Zhang et al., 2017). Meanwhile, the expression of Sirt3, NAD+, and NAMPT were reduced in the KLF4-deficient heart, all of which decreased deacetylase activity in the mitochondria. Moreover, NMN increased NAD+ levels and normalized the mitochondrial protein acetylation levels in cardiac tissue. Remarkably, NMN preserved the cardiac contractile function and protected CM-K4KO mice from heart failure during pressure overload. LCAD is a fatty acid oxidation (FAO) enzyme, which oxidizes long-chain fatty acid, the main fuel of heart. The activity of this enzyme is dependent on Sirt3-NAD+ deacetylation (Hirschey et al., 2010), and acute NMN treatment increased mitochondrial FAO, indicating that NMN improves cardiac energetics and heart function. CM-K4KO hearts showed high rates of cell death in myocardium when subjected to stress, and NMN administration prevented cell death in pressure-overloaded hearts. Finally, NMN was found to preserve the mitochondrial ultrastructure and reduce ROS and inflammation in CM-K4KO myocardium partially through Sirt3-dependent deacetylation and activation of SOD2 in response to oxidative stress (Zhang et al., 2017). In summary, short-term administration of NMN may confer protection against cardiac mitochondrial homeostasis and prevent heart failure.

Martin et al. (2017) reported that NMN improves cardiac function and bioenergetics in a SIRT3-dependent manner in a Friedreich’s ataxia (FRDA) cardiomyopathy model, a mouse genetic cardiomyopathy model with frataxin-KO (Fxn^–/–^, FXN-KO). NMN (500 mg/kg) administered two times per week for 4–5 weeks improves diastolic function and normalizes the defective cardiac contractility in FXN-KO. A shortened ejection time (ET) represents impaired contractility or severe LV dysfunction in patients with heart failure or idiopathic-dilated cardiomyopathy (Dujardin et al., 1998). FXN-KO mice show a shortened ET, whereas NMN treatment significantly increases ET (Martin et al., 2017).

NMN supplementation also improves cardiac energy utilization and decreases energy wastage in the FXN-KO heart. Cardiac efficiency (CE), a ratio of energy used for mechanical work to overall energy available, is decreased in FXN-KO. Also, FXN-KO mice also shows an increased ventriculoarterial coupling (VC) ratio, another parameter representing the efficiency of converting ventricular mechanical energy (ME) into hydraulic energy in the aorta during systole (Suga, 1990; Walley, 2016). NMN treatment normalizes both CE and VC in the FXN-KO heart failure model. Pressure–volume area (PVA) is the sum of ME and potential energy (PE) (Suga, 1990). In FXN-KO hearts, a large proportion of PVA is consumed as PE, rather than ME, which indicates greater energy wastage in heart failure. However, NMN treatment improves myocardial energy utilization by normalizing the proportion of PE in PVA.

Additionally, the study reported that NMN-induced reduction in energy wastage paralleled the remarkable decrease in whole-body energy expenditure (EE) due to decreased FA metabolism and a SIRT3-dependent carbohydrate metabolism (Martin et al., 2017). FXN-KO mouse show elevated whole-body daily FA oxidation and serum triacylglycerides, while NMN supplementation blocks this elevation. Moreover, NMN reduces tissue lactate production and inhibits glycolysis by decreasing systemic and cardiac glucose utilization in the FXN-KO heart, thereby improving CE and function (Martin et al., 2017). Importantly, the therapeutic effects of NMN are mediated by deacetylation of SIRT3 in FXN-KO hearts (Martin et al., 2017). In summary, these findings provide preclinical evidence that NMN could be a promising drug for heart failure and cardiomyopathies.

## Vascular Dysfunction

Cardiovascular disease (CVD) is the leading cause of mortality worldwide. Aging is a developmental risk factor for the disease. Vascular endothelial dysfunction and large elastic artery stiffness are two antecedents and predictors of clinical CVD (Mitchell, 2014). Vascular oxidative stress contributes to vascular endothelial dysfunction and large elastic artery stiffness (Bachschmid et al., 2013). Excessive superoxide reduces the bioavailability of nitric oxide (NO), a vasoprotective, and vasodilatory molecule, and thus causes alterations of structural proteins such as collagen and elastin, in large elastic arteries (Seals et al., 2014). Vascular endothelial dysfunction and large elastic artery stiffness are assessed by endothelium-dependent dilation (EDD) and aortic pulse wave velocity (aPWV), respectively (de Picciotto et al., 2016). Old mice showed impaired carotid artery EDD, and oral supplement of NMN (300 mg/kg) for 8 weeks restored EDD in the old mice by assisting in regaining NO-mediated dilation and reducing arterial oxidative stress (de Picciotto et al., 2016). Also, NMN restored the expression and activity of the SIRT1 in the arteries of old mice in accordance with previous studies, which revealed that the reduced expression and activity of SIRT1 contributes to impaired EDD in aging arteries (Donato et al., 2011; Gano et al., 2014) and by applying SIRT1 activator, the EDD was improved by partly reducing the oxidative stress (Gano et al., 2014).

An increase in stiffness of large elastic artery with age reduces the artery’s ability to buffer increase in pressure churned out by systolic ejection (de Picciotto et al., 2016). The type I collagen, a load-bearing protein in the arterial wall, is augmented during aging, while the main structural protein, elastin, diminishes in old arteries (Diez, 2007). Treatment using NMN also reduced the large elastic stiffness in aged mice by reversing the accumulation of whole-vessel type I collagen and enhancing arterial elastin (de Picciotto et al., 2016). A recent study showed that the protective effects of NMN supplement on vascular function are associated with increased anti-aging miRNA expression profile in the aorta of aged mice (Kiss et al., 2019b).

The brain, which is, a metabolically active organ, relies on the blood circulation to deliver nutrients and eliminate metabolic waste products (Tarantini et al., 2017). Cerebromicrovascular health is critical for brain perfusion, which helps in the maintenance of healthy cerebral function. Adjustment in cerebral blood flow maintains cellular homeostasis and function through neurovascular coupling (NVC), in response to the increased neuronal activity (Tarantini et al., 2017). Microvascular endothelium releases the vasodilator (NO) in response to increased neuronal and astrocytic activation (Toth et al., 2014). Several lines of evidence suggest that NVC responses are impaired in the course of aging, hence contributing to age-related cognitive impairment (Toth et al., 2014; Balbi et al., 2015). Emerging studies have revealed that an increase in mitochondrial oxidative stress and mitochondrial dysfunction results in impairment of neurovascular during the aging process (Springo et al., 2015).

A report by a recent study on aged mice showed that NMN could restore the function of cerebromicrovascular endothelium and NVC responses (Tarantini et al., 2019). NMN (i.p. 500 mg/kg) was injected in male aged mice (24 months old) for 14 consecutive days. The treatment restored the NO mediation of NVC in aged mice by reestablishing NO release, which resulted in endothelial NO-mediated vasodilation in the aortas of aged mice. The NO mediation of NVC responses is weakened by age-related mitochondrial oxidative stress. NMN increased the activation of SIRT1, which reversed mtDNA-encoded subunits, attenuated mtROS production, and improved mitochondrial bioenergetics. Thus, NMN can restore the protective effects of cerebromicrovascular by improving endothelial function, attenuating endothelial oxidative stress, and improving NVC responses in aged mice (Tarantini et al., 2019).

Age-related NAD+ depletion and consequential SIRT1 dysregulation are associated with impaired angiogenic processes in cerebromicrovascular. However, a study indicated that NMN could rescue angiogenic capacity in aged cerebromicrovascular endothelial cells (ECs) (Kiss et al., 2019a). Recently, the same team identified 590 genes differentially expressed in the aged neurovascular unit, 204 of which were shifted back toward youthful expression levels by NMN (Kiss et al., 2020). NMN supplementation reverses age-related changes in neurovascular gene expression, including SIRT1 activation, mitochondrial protection, anti-inflammatory, and anti-apoptotic (Kiss et al., 2020).

A steady decline in tissue perfusion has been observed in humans from the age of 40, which often results in organ dysfunction and general body weakness in the last decades of life (Le Couteur and Lakatta, 2010). The number and function of ECs decline with age, resulting in increased apoptosis of ECs in the muscle, diminished neovascularization, and loss of blood vessels (Wang et al., 2014). These changes reduce muscle mass and endurance during aging (Prior et al., 2016). Abhirup et al. reported that SIRT1 in ECs is necessary for the response to pro-angiogenic signals secreted from myocytes and treatment with NMN improved blood flow and endurance in elderly mice by increasing capillary density (Das et al., 2018). In the same study, the number and density of ECs and capillaries in skeletal muscle for endurance were significantly decreased in the old mice, which could have been associated with impaired angiogenesis. Endothelial-specific SIRT1 knockout mice (ESKO) exhibited the same phenotype of aging, including reduced density of capillaries and decreased exercise endurance. Overexpression of endothelial SIRT1 sensitizes ECs to vascular endothelial growth factor, which improves the muscle neovascularization and hence increases capillary density and endurance. Moreover, NAD+/SIRT1 negatively regulates Notch signaling (Guarani et al., 2011), an indispensable signaling pathway for blood vessel formation (Blanco and Gerhardt, 2013).

Thus, NMN could restore angiogenesis in old mice through SIRT1-mediated inhibition of Notch signaling. NMN (400 mg/kg/day) was administered to old mice through drinking water for 2 months. The drug restored the number and density of capillaries, increased soluble oxygen (sO_2_) levels, and improved endurance in the old mice. However, these effects were abolished in SIRT1 knockout mice. Also, exogenous hydrogen sulfide (H_2_S) augmented the effect of NMN.

All these studies suggest that NMN could be a favorable agent for therapy against various diseases related to a decrease in blood flow brought about by cardiovascular dysfunction.

## Intracerebral Hemorrhage

Intracerebral hemorrhage (ICH) is a primary brain injury resulting from mechanical damage that causes a hematoma. It can sometimes progress to a secondary injury, which is mainly subsequent pathophysiologic changes, including blood–brain barrier (BBB) destruction, hemoglobin-induced iron overload, neural cell death, neuroinflammation, and oxidative stress (Zhou et al., 2014; Wei et al., 2017b). A study suggested that NMN could be a promising agent for the treatment of ICH (Wei et al., 2017b). A single dose of NMN (300 mg/kg) was injected into CD1 mice 30 min after treatment with collagen-induced intracerebral hemorrhage (cICH). The NMN treatment relieved the edema that had been induced by cICH and improved neurological function. Treatment using NMN reduced cell death and oxidative stress in hemorrhage area. Moreover, it suppressed the cICH-induced microglia activation and neutrophil infiltration and inhibited inflammatory-associated factors, including TNF-α and IL-6. The intercellular adhesion molecule-1 (ICAM-1) protein, an adhesion molecule, is essential for the process of neuroinflammation activation after ICH. The NMN treatment also inhibited an increase in ICAM-1 after cICH (Sozzani et al., 2015; Wei et al., 2017b).

The secondary stage of ICH mainly results from toxicity caused by hemin, which is a breakdown product of hemoglobin. It causes cell death and induces extensive local inflammation/oxidative stress. Hemin can induce HO-1, a ubiquitous enzyme, which oxidatively cleaves pro-oxidant heme to produce biliverdin and carbon monoxide. NMN also elevates the nuclear Nrf2 protein expression to upregulate HO-1 protein expression in brain tissues, thereby inhibiting neuroinflammation and oxidative stress and contributing to the neuroprotection in ICH (Mylroie et al., 2015; Wei et al., 2017b). Finally, a prolonged NMN treatment for 7 days significantly arrested body weight decline and neurological deficit caused by ICH.

Carrie et al. also reported that NMN supplement preserved mitochondrial function, mitigated inflammation, and increased survival rate in hemorrhagic shock (Sims et al., 2018). Lactic acidosis, which is a by-product of anaerobic metabolism, is used to estimate the success of resuscitation in injured patients (Broder and Weil, 1964). High lactate levels reflect tissue hypoperfusion, which is, in turn, related to the severity and survivability of the shock (Rixen and Siegel, 2005). Pretreatment (400 mg/kg/day for 5 days, oral) and resuscitation (400 mg/kg, intravenous) with NMN significantly reduced serum lactate levels during a fixed pressure hemorrhagic shock.

The NAD+ concentration in various tissue decreases rapidly with the severity of the injury during hemorrhagic shock (Wurth et al., 1973), which can result in dysfunction of vital organs. Under hypoxic conditions, the mitochondrial electron transport chain becomes inefficient in reoxidizing NADH to the NAD (Wheaton and Chandel, 2011). The NMN treatment can compensate for the decrease in NAD+ levels and preserved the NAD/NADH ratio in both kidneys and livers. Adenosine triphosphate reserves are also depleted following hemorrhagic shock and resuscitation, and NMN treatment also restores the reserves in the kidney, but, not the liver. This could be caused by elevated ATP consumption in the liver. Treatment with NMN preserves the impaired complex I–dependent (CI-dependent) respiration following hemorrhagic shock and resuscitation.

Hemorrhagic shock and resuscitation are usually accompanied by increased cytokine levels, oxidative stress, and insulin-resistant hyperglycemia, which indicates the existence of inflammation (Sims et al., 2018). Circulating levels of the cytokine IL-6 are as predictors of mortality in human patients (Stensballe et al., 2009). Treatment with NMN significantly reduces serum IL-6 cytokine levels and ameliorated shock-induced hyperglycemia. The most important is that NMN can enhance physiologic reserve and improve survival after hemorrhagic shock. Pretreatment with NMN increases the ability of animals to tolerate longer periods of hypoperfusion. Resuscitation with NMN significantly improves the survival.

Tissue plasminogen activator (tPA) is used for therapy in case of acute brain ischemia. The window of tPA therapy is within 0–4.5 h. A delayed tPA therapy dose not decrease infarction but worsens hemorrhagic transformation (Wei et al., 2017a; Zhang et al., 2009). Wei et al. (2017a) suggested that NMN could attenuate delayed tPA-induced hemorrhagic transformation after cerebral ischemia without changing tPA thrombolytic activity. In the study, delayed tPA therapy resulted in a high mortality rate in middle cerebral artery occlusion (MCAO) mice. However, a single dose of NMN (300 mg/kg) injection (i.p.) reduced the mortality rate. NMN also attenuated the delayed tPA-induced severe infarction volume and brain edema. The cerebral hemorrhage caused by delayed tPA was fatal, but NMN significantly decreased the cerebral hemorrhage and higher hemoglobin level in the ipsilateral hemisphere of delayed tPA mice. The administration of NMN also inhibited neural apoptosis and ameliorated microglial activation and reduced neuroinflammation in delayed tPA-treatment mice.

It has been reported that disruption of BBB could be a reason for tPA-induced hemorrhagic transformation (Kastrup et al., 2008). The tPA activates matrix metalloproteinases (MMPs), which destroys tight junction proteins (TJPs) (Rempe et al., 2016). Consistent with previous findings, the protein levels of TJPs (claudins, occludin, and zonular occludens-1) were downregulated, and the activities of MMP9 and MMP2 were enhanced in delayed tPA treatment, which implied that the BBB integrity had been compromised. The NMN treatment significantly reversed these changes and protected BBB integrity. Thus, NMN could be a potential agent for the therapy of tPA-induced hemorrhagic transformation.

It was also reported that NMN is able to protect brain in both the early and the chronic phase of cryoinjury (Zhang et al., 2015). These studies demonstrated that NMN could be a remedy for ICH of various causes.

## Neuroprotective and Cognitive Function

Cognitive decline is one of the many symptoms of aging, and regulation of adult neurogenesis could be a therapeutic strategy in overcoming the condition. There are two distinct populations of neural stem cells (NSCs) in the brain, which are found in the subgranular zone (SGZ) and subventricular zone (SVZ). They can self-renew and differentiate into transient amplifying progenitors, that is, neural stem/progenitor cells (NSPCs). The NSPCs undergo finite, lineage-restricted cell divisions to differentiate into the major cell types of the brain, such as neurons, oligodendrocytes, and astrocytes (Artegiani and Calegari, 2012; Jadasz et al., 2012). Studies have revealed that aging is a negative regulator of NSPC proliferation, whereas NSPCs could be reactivated in the aged brain (Artegiani and Calegari, 2012). Thus, restoring the function of NSPCs could effectively prevent age-associated cognitive decline.

Liana and Shin-ichiro established that, in response to insult-induced demyelination, NAMPT-mediated NAD+ biosynthesis was critical for NSPC self-renewal, proliferation, and differentiation into oligodendrocytes (Stein and Imai, 2014). The study found that levels of NAD+ and expression of NAMPT in hippocampus were declining with age. However, the long-term NMN administration was able to maintain the NSPC pool. Moreover, they found that NMN administration reduced defects in oligodendrogenesis caused by decrease in NAD+ levels. Thus, NMN could be a promising agent for maintaining the NSPC pool and reactivating NSPCs, which can improve remyelination caused by aging and neurodegenerative diseases (Stein and Imai, 2014). Also, Zhao et al. (2015) reported that NMN was able to induce NSC proliferation (via SIRT1 and SIRT2) and promote NSC differentiation (via SIRT1, SIRT2, and SIRT6).

Mental disorders such as anxiety are prevalent among older people and account for 10–20% of the total mental diseases in the population, most of which is dementia or major depressive disorder (Regier et al., 1988). Late-life anxiety disorder results in substantial financial burden on individuals and society. Sean et al. discovered that old mice developed cognitive hypersensitivity in response to aversive stimulation during contextual fear condition tests, which was associated with the age-related changes in emotionality and sensory processing (Johnson et al., 2018). Moreover, specific knockdown of NAMPT in the hippocampal CA1 region recapitulated the same age-associated cognitive hypersensitivity, whereas dentate gyrus specific NAMPT knockdown mice did not show cognitive hypersensitivity.

Sean et al. found that calcium/calmodulin-dependent serine protein kinase (Cask), a crucial multidomain scaffold protein at the synapse and in cell junctions (Hsueh, 2006), is the downstream effector in response to the reduction in NAD+ and is also downregulated in the hippocampus during aging (Johnson et al., 2018). Therefore, Sean et al. proposed a model to demonstrate that the levels of NAMPT and NAD+ in hippocampal neurons, particularly in CA1 neurons, decline during aging, leading to a decrease in SIRT1 activity and subsequent downregulation of Cask expression in the aged hippocampus. Cask interacts with the GluN2B subunit of *N*-methyl-D-aspartate receptor (NMDAR) for the transport of GluN2B-containing vesicles (Jeyifous et al., 2009), and the reduction in Cask could cause dysfunction of GluN2B-containing NMDARs at the synapse, which could contribute to a wide range of cognitive and behavioral impairments during aging (Johnson et al., 2018). The NMN supplementation, even in a short time, could reduce cognitive hypersensitivity and improve the sensory processing aspect of some aversive stimuli and possibly other related behaviors (Johnson et al., 2018). Therefore, the NMN administration could prevent and treat such cognitive impairments and enhance the quality of life in old age.

NMN also prevented aging-induced cognitive impairment by improving cerebrovascular (Kiss et al., 2019a; Tarantini et al., 2019) and mitochondrial function, and reducing apoptosis in the prefrontal cortex and hippocampus of aged animals (Hosseini et al., 2019a). Lu et al. (2014) reported that NMN improved energy activity and survival rate in an *in vitro* model of Parkinson’s disease. Fang et al. (2016) also demonstrated that NR and NMN were able to normalize neuromuscular function and memory by regulation of mitophagy and enhancement of DNA repair in mice and worm models of ataxia-telangiectasia (AT), an autosomal recessive disease with progressive neurodegeneration.

A study indicated that NAMPT enzymatic activity enhancer P7C3 could have a neuroprotective effect in a mouse model of amyotrophic lateral sclerosis (ALS) (Tesla et al., 2012), and the levels of iNAMPT in ALS patients were lower than those of age-matched controls (Wang et al., 2017). Wang et al. (2017) silenced NAMPT in the projection neurons of adult mice and created Thy1-YFP-Nampt^–/–^ cKO mice that notably exhibited general motor abnormalities, muscle atrophy, progressive motor function deficits, and a shorter lifespan, some of which are the key features of ALS. Loss of NAMPT in projection neurons led to mitochondrial metabolic dysfunction and destroyed the mitochondrial homeostasis. Wang et al. reported that NAMPT knockdown disrupts the balance between mitochondrial fission/fusion and causes more fragmentation, which could lead to subsequent neuronal degeneration. The thy1-YFP-Nampt^–/–^ cKO mice exhibited neurodegeneration in the brain, especially in the motor cortex and increased acetylation proteins, which implied that NAD+-related perturbations induced a reduction in Sirt3 activity. Also, many lines of evidence have shown that increasing Sirt1 and Sirt3 deacetylation activities have a neuroprotective role in motor neuron degeneration diseases, such as ALS (Fu et al., 2012; Watanabe et al., 2014).

The thy1-YFP-Nampt^–/–^ cKO mice also showed widespread abnormalities of the neuromuscular junctions (NMJs), which is known to disrupt synaptic connectivity and cause defective synaptic transmission. Remarkably, NMN (400 mg/kg) treatment lessened disease severity, restored motor function, and extended the life span of Thy1-YFP-Nampt^–/–^ cKO mice. Recently, this same team reported that loss of NAMPT in projection neurons cause detrimental effects on the function and structure of NJM, including impaired synaptic vesicle cycling, morphological changes to the motor endplate, alterations of skeletal muscle contractile responses, and noticeable sarcomere misalignment (Lundt et al., 2020). These detrimental effects were reverse by administration of NMN (400 mg/kg/day) for 14 days. However, NMN treatment did not restore skeletal muscle mitochondria morphology change caused by deletion of Nampt (Lundt et al., 2020). Thus, NMN is a potential therapeutic drug for motor neuron (MN) degenerative diseases, including ALS. Increasing the NAD+ salvage pathway could reduce the symptoms of neurodegenerative diseases.

## Alzheimer’s Disease

AD is progressive dementia characterized by memory loss at an early stage of the disease progression. Currently, there is no effective therapy for AD treatment. Also, its molecular basis has not been elucidated (Gong et al., 2003). Thus, there is a need to focus on finding effective therapeutic drugs for the disease.

Mitochondrial dysfunction is a feature of many neurodegenerative diseases, including Alzheimer’s disease (AD), Parkinson’s disease (PD), and Huntington’s disease (HD) (Hroudova et al., 2014). Morphological and functional abnormalities of mitochondria may lead defects in the electron transport chain and ATP production, which are associated with AD (Hroudova et al., 2014; Long et al., 2015). The NAD+ as a cofactor is crucial for the tricarboxylic acid cycle, mitochondrial oxidative phosphorylation, and glycolysis-related enzymatic reactions, and the levels of NAD+ in the cell are critical for neuronal survival (Liu et al., 2008). Thus, preventing NAD+ depletion and boosting cellular energy could be a therapy for neurodegenerative disease (Long et al., 2015).

A study reported that NMN improved NAD+ catabolism and changed mitochondrial morphological dynamics in mice with AD (Long et al., 2015). In the study, AD chimeric amyloid precursor protein APP_(swe)_/PS1_(__Δ__E__9__)_ double transgenic (AD-Tg) mice showed deficits in mitochondrial oxygen consumption rates (OCRs) in the brain and muscle at 3 months of age. The NMN (100 mg/kg) was subcutaneously injected to AD-Tg female and male mice consecutively for 28 days, which reversed mitochondrial OCR deficiencies in the AD model. Still, in the same study, full-length mutant human APP levels were significantly increased in the brain of AD-Tg mice compared to non-transgenic (NTG) mice. The administration of NMN decreased full-length mutant APP expression in AD-Tg mice. Immunoreactivity of SIRT1 and CD38 was also significantly increased in AD-Tg compared to NTG mice, which could be attributed to NAD+ catabolism. The AD mice pre-treated with NMN showed a lower SIRT1 immunoreactivity compared to Ag-TD mice, but still had a higher SIRT1 compared to NTG mice.

The mitochondrial morphology is vital for its functions, such as mitochondrial respiration and calcium homeostasis, among others. Fusion and fission are two fundamental processes in mitochondria, which are essential for cellular survival and disease vulnerability. The protection of the nervous system is associated with the fusion, and fission processes, both of which contribute to the damaged organelles’ elimination (Escobar-Henriques and Anton, 2013). NMN administration increased the length of mitochondria and decreased fragmentation in the hippocampal sub-region (Long et al., 2015). A study reported that NMN alters mitochondrial dynamics by SIRT3-dependent mechanism (Klimova et al., 2019). Nina et al. also showed that the levels of the active form of fission protein, phosphorylated Drp1 (S616) (pDrp1), were significantly decreased in hippocampal mitochondria after a single-dose NMN treatment, and the same result was observed in AD-Tg mice (Long et al., 2015). NMN also decreased the acetylation of mitochondrial proteins, such as mitochondrial SOD2, which is an important antioxidant enzyme and one of the targets of SIRT3. The SOD2 catalyzes superoxide into hydrogen peroxide and oxygen, which is then converted to water by glutathione peroxidase (Chen et al., 2011). Studies have shown that mitochondrial morphologies are associated with the generation of ROS, and the accumulation of ROS could induce more mitochondrial fragmentation (Willems et al., 2015). Thus, NMN treatment reduced hippocampal ROS through SIRT3-dependent deacetylation of SOD2, which resulted in lowering mitochondrial fragmentation via pDrp1 (S616).

The Aβ oligomers have been widely reported to be responsible for AD pathology. They usually accumulate in AD frontal cortex levels of up to 70-fold compared to normal brains (Gong et al., 2003). Wistar rats with an intracerebroventricular infusion of Aβ_1__–__42_ oligomer were used as AD mouse models. Intraperitoneal NMN (500 mg/kg) treatment improved their cognitive and memory functions, which might have been as a result of NMN reducing oxidative stress actions (Wang et al., 2016). Long-term potentiation (LPT), a mechanism for memory and learning functions, is significantly inhibited by Aβ oligomers, but NMN treatment was able to prevent this inhibition in organotypic hippocampal slice cultures (OHCs). Oxidative stress and a high concentration of ROS have toxic effects on synaptic transmission, which is essential in neurodegeneration (Guidi et al., 2006). Administration of NMN prevented the accumulation of ROS induced by Aβ_1__–__42_ oligomer in OHCs, which could have been the reason for memory and learning improvement. The NMN treatment also attenuated the neuronal cell death and reduction of NAD+ and ATP in Aβ_1__–__42_ oligomer-treated hippocampal slices (Wang et al., 2016).

The c-Jun N-terminal kinases (JNKs) are a family of multifunction-signaling protein kinases that respond to various cellular stresses and inflammatory mediators (Mehan et al., 2011). However, aberrant activation of JNK is associated with pathogenesis of Alzheimer’s disease. A study demonstrated that NMN could reverse AD by inhibiting JNK activation in the hippocampus and cerebral cortex of AD-Tg mice (Yao et al., 2017). Administration of NMN ameliorated amyloid-induced synaptic loss and dysfunction, and reversed cognitive impairments in AD-Tg mice, including severe impairment of spatial learning, spatial memory and contextual memory.

Also, NMN decreased β-amyloid production and amyloid plaque burden (Yao et al., 2017). The NMN regulated the expression of APP cleavage secretase in AD-Tg mice, including elevation of sAPPα and reduction of sAPPβ. A previous study indicated that phosphorylation of APP on threonine 668 could promote β-secretase cleavage of APP, resulting in more Aβ generation (Colombo et al., 2009). SIRT1 activation promoted non-amyloidogenic α-secretase processing of the amyloid precursor protein by inhibiting rho-associated kinase (ROCK1) expression (Qin et al., 2006). These results showed that NMN might enhance non-amyloidogenic APP processing and hence decrease Aβ pathology in AD-Tg mice. Neuroinflammatory, a crucial response for the development of AD (Morales et al., 2014), was shown to be inhibited by NMN treatment in AD-Tg mice (Yao et al., 2017), including decrease in IL-6, IL-1β, and TNFα. These proinflammatory cytokines stimulate β-secretase and γ-secretase to generate more Aβ in the brain through a JNK-dependent MAPK pathway (Liao et al., 2004). In conclusion, NMN improved cognitive abilities and decreased amyloid plaque, loss of synapse, β-secretase, and neuroinflammation, at least partially by inhibition of JNK activation.

These studies demonstrate that NMN could be a viable intervention for managing AD.

## Retinal Degeneration and Corneal Injury

The photoreceptor is crucial for light transduction, which is necessary for vison, and there are two classes of photoreceptors: rods and cones. Rod and cone photoreceptors mediate dim and precise central vision in ambient light, respectively (Lin et al., 2016). Death of photoreceptors results in vision loss, and this occurs in many diseases, including age-related macular degeneration (AMD), retinitis pigmentosa (RP), and Leber congenital amaurosis (LCA) (Wright et al., 2010). It has been reported that mutations in NMNAT1 cause LCA, as a result of reduced NAD+ biosynthesis and impaired protein folding (Koenekoop et al., 2012). Thus, NAD+ biosynthesis has a role in photoreceptor function and survival.

Knockout mice lacking Nampt in rod photoreceptors (Nampt^–rod/–rod^) and cone photoreceptors (Nampt^–cone/–cone^) showed a decreased retinal NAD+ level and a degenerative phenotype, including vascular attenuation, optic nerve atrophy, outer nuclear layer thickness reduction, and retinal function impairment (Lin et al., 2016). Retinal NAD+ deficiency was observed in multiple mouse models with retinal dysfunction, including light-induced degeneration, streptozotocin (STZ)-induced diabetic retinopathy, and aging-associated retinal dysfunction. NAD+ deficiency in photoreceptors causes significant glycolytic and mitochondrial dysfunction under basal conditions and impairs the normal response to moderate metabolic stresses, which results in photoreceptor cell death and retinal degeneration (Lin et al., 2016). Besides, photoreceptors are susceptible to defects in energy homeostasis because of their limited mitochondrial reserve (Kooragayala et al., 2015). Lin et al. (2016) demonstrated that SIRT3 and SIRT5 are both critical for photoreceptor survival and retinal homeostasis. It is through SIRT3 that NAD+ deficiency causes aberrant hyperacetylation of mitochondrial proteins, hence contributing to mitochondrial dysfunction.

Nampt is critical for energy metabolism in retinal cells and its deficiency results in impairment of retinal homeostasis. Downstream production of Nampt is a potential approach to relieve the impairment. Intraperitoneal injections of NMN (150 mg/kg) for 4 weeks improved scotopic and photopic retinal function and reduced photoreceptor death in Nampt^–rod/–rod^ and Nampt^–cone/–cone^ mice. Also, intraperitoneal injections of NMN (300 mg/kg) for 10 days were able to protect the retina from light-induced injury (Lin et al., 2016).

Mills et al. reported that long-term NMN administration ameliorated age-associated pathological changes in the eyes (Mills et al., 2016). The mutation in rd8 of C57BL/6N mice induced accumulation of subretinal microglia and macrophages with age, which was consistent with an increase in light-colored spots in the fundus of the eyes (Aredo et al., 2015; Mills et al., 2016). The C57BL/6N mice at 17 months of age showed several light-colored spots in the fundus. However, the spots were significantly reduced in the fundus of the aged mice, which were on a long-term NMN supplementation. The long-term supplementation prevented rod cell dysfunction and improved scotopic b and photopic b waves in aged C57BL/6N, which suggested that the function of Muller/bipolar cell and cone cell was enhanced by NMN supplementation (Mills et al., 2016). The functioning of the lacrimal gland in humans and rodents gradually decreases with age (Zoukhri, 2006). Notably, long-term NMN administration significantly increased tear production in aged mice (Mills et al., 2016).

The cornea is one of the most densely innervated tissues in the human body and corneal innervations play a critical role in the regulation of epithelial homeostasis (Bonini et al., 2003). Neurotrophic keratopathy, a degenerative corneal disease with an impairment of trigeminal nerve, shows corneal epithelial defects, ulcer, and even perforation (Bonini et al., 2003). Li et al. demonstrated that corneal denervation impaired the epithelial NAD+ levels by reducing the expression of NAMPT. This process led to deactivation of SIRT1, pAKR, and pCREB, and caused the apoptosis of corneal epithelial cells (Li et al., 2019). NMN treatment significantly reduced the wound area and slowed down the corneal nerve fiber degeneration in the denervated mice. The supplement of NMN restored the activation levels of SIRT1, AKT, and CREB, and reversed the cell apoptosis and epithelial defects (Li et al., 2019). A recent study also reported that subconjunctival injection of NMN or other NAD+ precursors effectively prevented ultraviolet B light (UVB)-induced tissue damage and EC apoptosis in the mouse cornea through reactivating AKT signaling (Zhao et al., 2020). These findings demonstrate that NMN could be a therapeutic agent for treating diverse diseases that are associated with blindness.

## Acute Kidney Injury (AKI)

Approximately 1.33 million people worldwide suffer from AKI every year, resulting in a substantial economic and social burden on patients and society. The condition is associated with a high mortality rate exceeding 50% of those affected and the development of chronic kidney disease and other types of organ dysfunctions (Lewington et al., 2013). Aging is an independent risk factor for AKI (Kane-Gill et al., 2015), and various age-related factors contribute to the increased susceptibility to AKI (Fan et al., 2013), including diabetes, hypertension, vascular diseases, and some iatrogenic factors (Kane-Gill et al., 2015).

Studies have revealed that SIRT1 and SIRT3 are critical in protecting the kidney from injury (Fan et al., 2013; Morigi et al., 2015). Guan et al. reported that the administration of NMN could prevent age-associated susceptibility to AKI by restoring renal SIRT1 activity (Guan et al., 2017). The study found that kidneys of aged mice were more susceptible to cisplatin-induced AKI, and the NAD+ levels and SIRT1 expression were low in aged kidney. NAMPT and NMNAT were significantly lower in the kidney cortex of the old mice compared with those of the young mice. Four days of NMN treatment restored NAD+ levels in the kidneys and protected them from age-associated AKI. However, this protective effect of NMN was inhibited by SIRT1 deficiency, indicating that the renal protective effect of NMN was dependent on SIRT1. NMN treatment also protects the kidney from ischemia–reperfusion injury. Mice with NMN administration showed less ischemia–reperfusion injury and better kidney function compared with mice treated with PBS, which includes decreased blood urea nitrogen (BUN) and lower serum creatinine levels, and ameliorated tubular damage (Guan et al., 2017). Moreover, it has been shown that NMN could alleviate diabetic nephropathy nephritic fibrosis by inhibiting endogenous Nampt (Chen et al., 2017).

In conclusion, the findings of these studies show that NMN could be a potential therapeutic agent for AKI because of its ability to restore NAD+ and SIRT levels in the kidney.

## Alcoholic Liver Disease

As the most common chronic liver disease, alcoholic liver disease (ALD) is caused by chronic alcohol consumption and can develop from alcoholic fatty liver (AFL) to alcoholic steatohepatitis (ASH) (Seitz et al., 2018). Ethanol-induced NAD+ depletion is involved in the development of ethanol-induced steatosis, oxidative stress, steatohepatitis, and insulin resistance (Luo et al., 2017). However, NMN treatment maintained the NAD+ levels and restored the alterations of TCA cycle metabolites that is induced by ethanol (Assiri et al., 2019). NMN also successfully prevented an ethanol-induced increase in plasma levels of alanine aminotransferase (ALT) and aspartate aminotransferase (AST), two damage biomarkers of liver. RNA-seq analysis revealed that ethanol changed the expression of 1778 genes, 25% of which were altered by NMN treatment (Assiri et al., 2019). The mitogen-activated protein kinases (MAPK) pathway was one of the signaling pathways that were significantly affected by NMN. Previous studies have demonstrated that activating transcription factor 3 (Atf3) is associated with NAD+/NADH ratios and can be induced by ethanol (Mohammadnia et al., 2015). Elevated Atf3 also correlated with increased ALT and AST (Allen-Jennings et al., 2002). Moreover, Atf3 overexpression has been found in patients with alcoholic steatohepatitis (Mohammadnia et al., 2015). Interestingly, NMN normalized extracellular signal-regulated kinase1/2 (Erk1/2) signaling and reduced the expression of Atf3 (Assiri et al., 2019).

## Other Diseases and Improvement of Physical Function

Associated with the loss of oocyte quality, reproductive aging in female mammals is an irreversible process that accompanies decreased levels of NAD+ (Bertoldo et al., 2020). However, administration of NMN can hopefully restore oocyte quality and fertility in aged mice and reverse the adverse effect of maternal age on developing embryo, suggesting that NMN can rescue female reproductive function in mammals (Bertoldo et al., 2020). Multigenerational obesity-induced perturbations in oocyte-secreted factor signal can also be normalized by NMN supplementation (Bertoldo et al., 2018). However, NMN does not protect the ovarian reserve from radiotherapy and chemotherapy, such as γ-irradiation or cyclophosphamide (Stringer et al., 2019).

Depression is a major mental health problem and has especially large effects on individual health and social burden. Previous studies have suggested that mitochondrial dysfunction and decreased ATP production contribute to depression (Allen et al., 2018). Recently, Xie et al. reported that in a corticosterone (CORT)-induced depressed mouse model, NMN could alleviate depression-like behaviors by improving mitochondrial energy metabolism via enhancing the activity of SIRT3 (Xie et al., 2020). Transcriptome and metabolome analysis demonstrated that NMN inhibited CORT-induced lipid synthesis, stimulated β-oxidation and glycolysis, and improved the TCA cycle to enhance ATP production in mitochondria.

**FIGURE 4:**
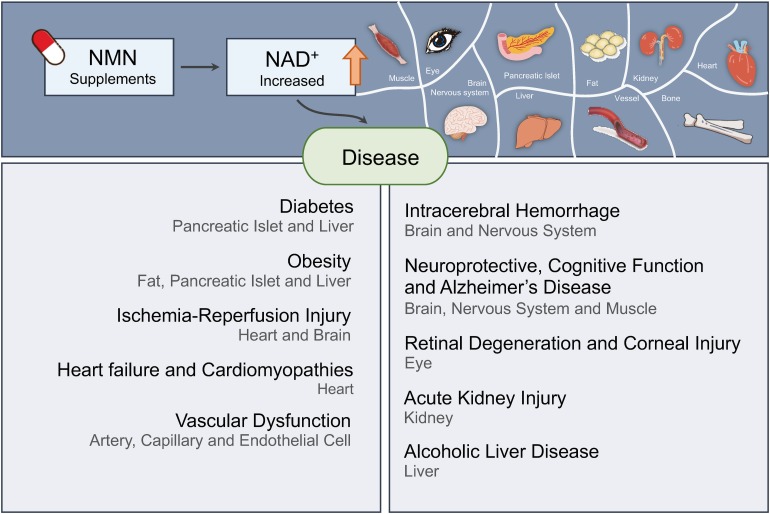
Nicotinamide mononucleotide ameliorates various diseases by increasing NAD+ levels in human. NMN is a promising molecule for therapy of diverse diseases, including diabetes, obesity, ischemia–reperfusion injury, heart failure, Alzheimer’s disease, retinal degeneration, acute kidney injury, and so on.

Besides, NMN has also been reported to enhance skeletal muscle mitochondrial oxidative metabolism in aged mice (Gomes et al., 2013), improve hepatic mitochondrial function in circadian mutant mice (Peek et al., 2013), reduce DNA damage, and protect against irradiation-induced alterations in white blood cell counts, lymphocytes, and hemoglobin (Li et al., 2017). Recently, a study reported that NMN treatment improved mesenchymal stromal cells self-renewal with promoted osteogenesis and reduced adipogenesis via the SIRT1 pathway in aged and irradiated adult mice (Song et al., 2019). Moreover, NMN supplement can alleviate aluminum-induced bone injuries via suppression of the thioredoxin-interacting protein (TXNIP)–NLRP3 inflammasome pathway (Liang et al., 2019). These findings further demonstrate that NMN can be used to treat a variety of diseases involving NAD+ decline (Figure 4 and Table 1).

**TABLE 1:** Therapeutic effects of NMN administration *in vivo.*

Disease	Model	Intervention	Effects	References
**Diabetes**	Nampt-deficient heterozygous (Nampt ^+/–^) mice	NMN (i.p. 500 mg/kg) Single	Improved the defects in NAD biosynthesis and glucose-stimulated insulin secretion	Revollo et al., 2007
	Adenoviral-mediated hepatic overexpression of miR-34a in mice	NMN (i.p. 500 mg/kg) 10 days	Improved glucose tolerance and increased expression of fatty acid β-oxidation genes	Choi et al., 2013
	Fructose-rich diet (FRD) mice	NMN (i.p. 500 mg/kg) Single	Improved insulin secretion and protected against inflammation	Caton et al., 2011
	High-fat diet (HFD)-induced and age-induced diabetic mice	NMN (i.p. 500 mg/kg) HFD-induced diabetes (7–10 days) aged-induced diabetes 11 days	Improved glucose tolerance and enhanced hepatic insulin sensitivity in HFD-induced diabetic mice. Improved glucose tolerance and lipid profiles in aged-induced diabetic mice	Yoshino et al., 2011
	C57BL/6N mice	NMN (drinking water. 100, 300 mg/kg) 12 months	Improved age-associated decreased insulin sensitivity and plasma metabolism	Mills et al., 2016
	Aged beta cell-specific Sirt1 overexpression (BESTO) mice	NMN (i.p. 500 mg/kg) Single	Improved glucose tolerance and insulin secretion in the aged BESTO females	Ramsey et al., 2008
Obesity	Adipocyte-specific Nampt knockout (ANKO) mice	NMN (drinking water. 500 mg/kg) 4–6 weeks	Improved multi-organ insulin sensitivity, increased adiponectin production and normalized plasma FFA concentrations	Stromsdorfer et al., 2016
	Adipocyte-specific Nampt knockout (ANKO) mice	NMN (drinking water. 500–1000 mg/kg) 8 weeks	Restored thermogenesis in ANKO mice, and normalized the expression of genes involved in thermogenesis, mitochondrial biogenesis, and FFA metabolism	Yamaguchi et al., 2019
	High-fat diet (HFD)-induced obese mice	NMN (i.p. 500 mg/kg) 17 days	Improved glucose tolerance, liver citrate synthase activity, and reduced liver triglyceride content	Uddin et al., 2016
	HFD-consuming offspring of obese mothers	NMN (i.p. 500 mg/kg) 18 days	Reduced adiposity and improved glucose tolerance and mitochondrial function	Uddin et al., 2017
	C57BL/6N mice	NMN (drinking water. 100, 300 mg/kg) 12 months	Reduced age-associated body weight gain	Mills et al., 2016
Ischemia–reperfusion injury	Ischemia/reperfusion heart injury in C57BL/6J mice	NMN (i.p. 500 mg/kg) Once or 4 times	Reduced the infarct area and improved left ventricle (LV) systolic function after I/R	Yamamoto et al., 2014
	Global cerebral ischemia in C57BL/6 mice	NMN (i.p. 31.25, 62.5, 125, 250, and 500 mg/kg) Single	Reduced hippocampal CA1 neurons cell death and improved ischemia-induced hippocampal dysfunction involved in spatial working memory (62.5 mg/kg produced optimal treatment effects)	Park et al., 2016
	Global cerebral ischemia in C57BL/6 mice	NMN (i.p. 62.5 mg/kg) Single	Prevented post-ischemic depletion of mitochondrial NAD+, suppressed mitochondrial fragmentation, and reduced ROS generation	Klimova et al., 2020
	Middle cerebral artery occlusion (MCAO) in Sprague-Dawley rats	NMN (i.c.v. 10 mg/ml with 2 μl) Single	Alleviated cerebral infarction size, neurological deficit, and neuronal cell death	Wang P. et al., 2011
	Middle cerebral artery occlusion (MCAO) in C57BL/6J mice	NMN (i.p. 500 mg/kg) 7 days with the first dose at 3 h (early) or 12 h (delayed) post MCAO	Alleviated brain infarction and neurological deficit, increased animal survival, and accelerated body weight recovery (early NMN administration). Improved post-ischemic regenerative neurogenesis (delayed NMN administration)	Zhao et al., 2015
Heart failure and cardiomyopathies	Cardiac-specific deletion of Ndufs4 (cKO) in mice	NMN (i.p. 500 mg/kg) twice in 3 days	Decreased the NADH/NAD+ ratio and mitochondria protein acetylation in cKO hearts, and normalized the sensitivity of the mitochondria permeability transition pore (mPTP)	Karamanlidis et al., 2013
	Transverse aortic constriction (TAC)-stressed mice, cKO mice	NMN (i.p. 500 mg/kg) 33 days (every 3 days)	Suppressed mitochondrial protein hyperacetylation, improved cardiac function, and reduced pathologic hypertrophy induced by pressure overload	Lee et al., 2016
	TCA in cardiac-specific deficiency of Klf4 (CM-K4KO) mice	NMN (i.p. 500 mg/kg) 5 days	Protected mice from pressure overload-induced heart failure, prevented cell death, preserved mitochondrial ultrastructure and reduced ROS in heart	Zhang et al., 2017
	Cardiac-specific Fxn KO mice (FXN-KO) (Friedreich’s ataxia cardiomyopathy model)	NMN (i.p. 500 mg/kg) 4–5 weeks (twice weekly)	Improved diastolic function, normalized the defective cardiac contractility, improved cardiac energy utilization and decreased energy wastage and whole-body energy expenditure in FXN-KO mice	Martin et al., 2017
Vascular dysfunction	Aged (26–28 months) C57Bl/6 mice	NMN (drinking water. 300 mg/kg) 8 weeks	Improved artery endothelium-dependent dilation (EDD) and NO-mediated EDD, reduced arterial oxidative stress and large elastic artery stiffness	de Picciotto et al., 2016
	Aged (24-month-old) C57BL/6 mice	NMN (i.p. 500 mg/kg) 14 days	Rescued neurovascular coupling (NVC) responses, and increased endothelial NO-mediated vasodilation	Tarantini et al., 2019
	Aged (24-month-old) C57BL/6 mice	NMN (i.p. 500 mg/kg) 14 days	Reversed age-related changes in miRNA expression profile in the aged mouse aorta	Kiss et al., 2019b
	Aged (24-month-old) C57BL/6 mice	NMN (i.p. 500 mg/kg) 14 days	Reversed age-related changes in neurovascular gene expression, including SIRT1 activation, mitochondrial protection, anti-inflammatory, and anti-apoptotic	Kiss et al., 2020
	Aged (18-month-old) C57BL/6J mice	NMN (drinking water. 400 mg/kg) 2 months	Improved blood flow and endurance in elderly mice by increasing capillary density	Das et al., 2018
Intracerebral hemorrhage	Collagenase-induced intracerebral hemorrhage (cICH) in CD1 mice	NMN (i.v. 300 mg/kg) Single (acute) NMN (i.v. and i.p. 300 mg/kg) 7 days (prolonged)	Relieved the edema, improved neurological function, reduced cell death and oxidative stress, and inhibited neuroinflammation in cICH. Promoted the recovery of body weight and neurological function (prolonged administration)	Wei et al., 2017b
	Hemorrhagic shock in Long–Evans rats	NMN (400 mg/kg) Pretreatment (drinking water) for 5 days and during resuscitation with once (i.v.)	Inhibited inflammation, improved cellular metabolism, and promoted survival following hemorrhagic shock.	Sims et al., 2018
	Tissue plasminogen activator (tPA)-treated CD1 mice with MCAO	NMN (i.p. 300 mg/kg) Single	Prevented delayed tPA-induced brain damage, cerebral hemorrhage, neural apoptosis, and neuroinflammation, and protected blood–brain barrier integrity	Wei et al., 2017a
Neuroprotective and cognitive function	C57BL/6N mice	NMN (drinking water, 100, 300 mg/kg) 12 months	Maintained the neural stem/progenitor cells (NSPCs) pool	Stein and Imai, 2014
	Aged (20-month-old) C57BL/6 mice	NMN (p.o. 300 mg/kg) 3 weeks	Improved cognitive hypersensitivity (age-related changes in sensory processing and emotionality) in old mice	Johnson et al., 2018
	Aged (24-month-old) C57BL/6 mice	NMN (i.p. 500 mg/kg) 14 days	Improved cognitive function in aged mice	Tarantini et al., 2019
	Aged (24-month-old) Wistar rats	NMN (i.p. 100 mg/kg) every other day for 28 days	Alleviated aging-induced cognitive impairment, improved learning and memory in aged animals	Hosseini et al., 2019a
	Projection-neuron-specific and inducible Nampt conditional knockout (Thy1-YFP-Nampt^–/–^ cKO) mice	NMN (i.p. 400 mg/kg) Started on day 11 post- tamoxifen administration	Alleviated disease severity, restored motor function, and prolonged the lifespan	Wang et al., 2017
	Thy1-YFP-Nampt^–/–^ cKO mice	NMN (i.p. 400 mg/kg) 14 days	Reversed the detrimental effects on vesicle cycling, endplate morphology and muscle contractility	Lundt et al., 2020
	Ataxia-telangiectasia mutated (ATM)-deficient mice (Atm^–/–^ mice) (Ataxia telangiectasia mouse model)	NMN (drinking water, 12 mM) 2 weeks	Restored deficit in motor function and improved memory in Atm^–/–^ mice	Fang et al., 2016
Alzheimer’s disease	APP_(swe)_/PS1_(__Δ__E__9__)_ double transgenic (AD-Tg) mice	NMN (s.c. 100 mg/kg) every other day for 28 days	Increased mitochondrial function and decreased amyloid precursor protein (APP) expression	Long et al., 2015
	C57BL/6 mice and neuron-specific expression of mitochondria-targeted enhanced yellow fluorescent protein (mito-eYFP) transgenic mice	NMN (i.p. 62.5 mg/kg) Single	Inhibited mitochondrial fission, decreased mitochondrial protein acetylation, and reduced ROS in the hippocampus	Klimova et al., 2019
	Intracerebroventricular infusion of Aβ_1__–__42_ oligomer in Wistar rats	NMN (i.p. 500 mg/kg) 10 days	Improved cognitive function	Wang et al., 2016
	APP_(swe)_/PS1_(__Δ__E__9__)_ double transgenic (AD-Tg) mice	NMN (s.c. 100 mg/kg) every other day for 28 days	Improved cognitive abilities and decreased amyloid plaque, loss of synapse, β-secretase, and neuroinflammation	Yao et al., 2017
Retinal degeneration and corneal injury	Rod-specific Nampt KO mice (Nampt^–rod/–rod^) Cone-specific Nampt KO mice (Nampt^–cone/–cone^) Light-induced retinal dysfunction (129S1/SvlmJ mice)	NMN (i.p. 150 mg/kg) 4 weeks for Nampt^–rod/–rod^ and Nampt^–cone/–cone^ mice NMN (i.p. 300 mg/kg) 10 days for 129S1/SvlmJ mice	Prevented photoreceptor degeneration and improved vision in Nampt^–rod/–rod^ and Nampt^–cone/–cone^ mice. Protected the retina from light-induced injury in 129S1/SvlmJ mice	Lin et al., 2016
	C57BL/6N mice	NMN (drinking water, 100, 300 mg/kg) 12 months	Ameliorated age-associated pathological changes in the eyes, and increased tear production in aged mice	Mills et al., 2016
	Corneal denervation in C57BL/6 mice	NMN (251 ng/eye) subconjunctival injection 2 days	Reduced wound area and slowed down the corneal nerve fibers degeneration in the denervated mice	Li et al., 2019
	Ultraviolet B light (UVB)-induced injury in C57BL/6 mice	NMN (500 mM, 5 μl/eye) subconjunctival injection 2 days	Prevented ultraviolet B light (UVB)-induced tissue damage and endothelial cell apoptosis in the mouse cornea	Zhao et al., 2020
Acute kidney injury (AKI)	Cisplatin-induced AKI or ischemia reperfusion injury in 129S2/Sv mice, and C57BL/6 mice	NMN (i.p. 500 mg/kg) 4 days	Protected renal function from cisplatin-induced AKI and ischemia reperfusion injury	Guan et al., 2017
Alcoholic liver disease (ALD)	Lieber–DeCarli chronic ethanol model in C57BL/6 J mice	NMN (i.p. 500 mg/kg) every other day for 6 weeks	Prevented an ethanol-induced increase in plasma alanine aminotransferase (ALT) and aspartate aminotransferase (AST), and changed the gene expression that were modulated by ethanol	Assiri et al., 2019
Other diseases	C57BL6/JAusb mice	NMN (drinking water, 0.5 and 2 g/L) for 4 weeks	Restored oocyte quality and enhanced ovulation rate and fertility in aged mice	Bertoldo et al., 2020
	HFD-consuming offspring of obese mothers	NMN (i.p. 500 mg/kg) 18 days	Ameliorated multigenerational obesity-induced perturbations in oocyte-secreted factor signal	Bertoldo et al., 2018
	Corticosterone (CORT)-induced depression in C57BL/6 mice	NMN (p.o. 300 mg/kg) 2 weeks	Alleviated depression-like behaviors	Xie et al., 2020
	C57BL/6J	NMN (i.p. 500 mg/kg) 7 days	Enhanced skeletal muscle mitochondrial oxidative metabolism in aged mice	Gomes et al., 2013
	Bmal1 KO mice	NMN (i.p. 250 mg/kg). Single NMN (i.p. 500 mg/kg) 10 days	Increased hepatic mitochondrial respiration	Peek et al., 2013
	Old C57BL/6J mice and young irradiated C57BL/6J mice	NMN (i.p. 500 mg/kg) 7 days. NMN (p.o. 2000 mg/kg) 8 days	Reduced DNA damage and protected against irradiation-induced changes in white blood cell counts, lymphocytes, and hemoglobin	Li et al., 2017
	Aged C57BL/6J (12-month-old) mice, and sublethal irradiation in adult (2-month-old) C57BL/6J mice	NMN (drinking water. 300 mg/kg) 3 months	Stimulated osteogenesis of endogenous mesenchymal stromal cells (MSCs), and protected bone from aging and irradiation induced damage in mice	Song et al., 2019
	AlCl_3_-treated Sprague–Dawley rats	NMN (i.p. 20 mg/kg) 4 weeks	Alleviated aluminum-induced bone loss	Liang et al., 2019
	Brain cryoinjury in Balb/c mice	NMN (i.c.v. 5 mM with 7 μl) Single	Protected brain in both the early and the chronic phase of cryoinjury	Zhang et al., 2015
	Adipocyte-specific Nampt knockout (ANKO) mice	NMN (i.p. 500 mg/kg) Single	Restored physical activity in ANKO mice	Yoon et al., 2015

*IP, intraperitoneal; IV, intravenous; PO, per oral; SC, subcutaneous; ICV, intracerebroventricular.*

## Possible Detrimental Effects of NMN Supplementary

Although the benefits of NMN are obvious, some studies suggest that people need to use it with caution. Keeping NMN at a low level was beneficial to the axon survival (Di Stefano et al., 2017). NMN accumulation could promote Wallerian or Wallerian-like degeneration, which was involved with the physical neurite injury. Inhibiting NAMPT or maintaining NMNAT activity could preserve the structure and function of the transected axons and the distal axon stump by preventing the increase of NMN. It is worth noting that since a long-term NAD+ depletion would become harmful for protection of axons, a time window of inhibiting the NMN accumulation was shown after the acute injury (Di Stefano et al., 2015). To decrease the level of NMN and maintain the production of NAD+, nicotinic acid riboside (NAR) was introduced in treating chemotherapy-induced axon degeneration by generating NAD+ through an alternative pathway that bypassed NMN formation (Liu et al., 2018).

It was reported that a lower dosage of NMN could be more effective and safer, for example, the brain may be more sensitive to the concentration of NMN (Park et al., 2016). A lower-dose administration of NMN resulted in improved female infertility with enhanced oocyte quality. Overdose of NMN may have some adverse effects on other aspects of fertility (Bertoldo et al., 2020). NMN administered by intraperitoneal injection increased oxidative stress of sperm and reduced sperm quality in male offspring of obese mothers with HFD, whereas oral administration of NMN did not have these effects (Youngson et al., 2019). These data indicated that the effects of NMN were complex and the therapeutic dose or mode of administration needs more investigation in the future.

Carefully, NAD turnover in tumor cells is higher than nontumor cells. The expression levels of NAMPT were found to be elevated and have a positive relationship with the stage of tumor progression in many types of tumor, indicating that downregulation of NAMPT/NAD+ may become a strategy for anti-cancer therapy (Wang B. et al., 2011; Gujar et al., 2016). It is noteworthy that SIRT1, as downstream targets, has both tumor-suppressor and oncogenic roles under different circumstances (Chalkiadaki and Guarente, 2015). Thus, efficiency and long-term safety of NMN should be precisely assessed in further preclinical and clinical studies.

## Human Clinical Study

Given that NMN has shown high efficacy and benefits in various mouse models of human disease, several clinical trials of NMN have been conducted to investigate its clinical applicability (Table 2). This has led to some capsule formulations of NMN being approved and put on the market as health supplements (Poddar et al., 2019).

**TABLE 2:** Human clinical trials of NMN.

Molecule	Objectives	Subjects and sample size	Intervention	Study design	Region and Institute	Phase	Trial number
NMN	Evaluate the safety and kinetics of NMN in healthy volunteers	Healthy men 40–60 years (*n* = 10)	NMN P.O. single time	Non-randomized non-label uncontrolled	Keio University School of Medicine in Japan	I	UMIN000021309
NMN	Evaluate the safety and kinetics of long-term NMN and its effect on glucose metabolism in healthy volunteers.	Healthy men 40–60 years (*n* = 30)	NMN P.O. 8 weeks	Non-randomized non-label uncontrolled	Keio University School of Medicine in Japan.	II	UMIN000030609
NMN	Evaluate the effect of the dietary (NMN) on key cardiovascular and metabolic functions in healthy women.	Postmenopausal women pre-diabetic BMI 25.0–44.9 55–75 years (*n* = 25)	Placebo or 250 mg/day NMN P.O. 8 weeks	Randomized double-blinded placebo-controlled	Washington University School of Medicine in United States of America	Active, not recruiting	NCT03151239
NMN	Evaluate the safety and effect of long-term NMN on various hormonal levels in healthy human.	Healthy men and women 50–70 years (*n* = 20)	100 or 200 mg/day NMN P.O. 24 weeks	Randomized double-blinded dose comparison	Hiroshima University in Japan	Not applicable	UMIN000025739
NMN	Evaluate the effect of NMN on the body composition in the elder	Healthy men no smoking BMI 22–28 ∼65 years (*n* = 42)	Placebo or 250 mg/day NMN P.O. 12 weeks	Randomized double-blind placebo-controlled	The University of Tokyo Hospital in Japan	Not applicable	UMIN000036321

The first phase I human clinical study (UMIN000021309) for NMN has been initiated by an international collaborative team between Keio University School of Medicine in Tokyo and Washington University School of Medicine in St. Louis. The aim of this study is to examine the safety and bioavailability of NMN in human bodies. This research is led by Hiroshi Itoh, a professor of Endocrinology, Metabolism and Nephrology, Tokyo and Shin-ichiro Imai, a professor of Developmental Biology, at Washington University (Tsubota, 2016). Recently, they reported that a single oral administration of NMN up to 500 mg was safe and effectively metabolized in healthy subjects without causing severe adverse events. The major final metabolites of NMN were significantly increased in a dose-dependent manner by NMN administration. However, the team failed to detect NMN in plasma samples in this study. In a further study, they will detect the levels of NMN in plasma and NAD+ in peripheral blood mononuclear cells (Irie et al., 2019).

Itoh is also conducting a phase II study (UMIN000030609) to assess the safety of long-term NMN in healthy subjects, the kinetics of NMN and metabolites of NAM, and the effect of daily administration of NMN on glucose metabolism. Other clinical trials of NMN are ongoing at Washington University to examine the effect of NMN on insulin sensitivity, endothelial function, blood lipids, body fat and liver fat, and fat tissue and muscle tissue markers of cardiovascular and metabolic health. Additionally, a study (UMIN000025739) has been initiated at Hiroshima University, Institute of Biomedical and Health Sciences to evaluate the effect of long-term oral administration of NMN on various hormones in healthy volunteers. Recently, a new clinical study (UMIN000036321) was initiated at the University of Tokyo Hospital to evaluate the effect of NMN oral administration on the body composition in elderly persons.

In summary, despite the tremendous research efforts aimed at exploiting the therapeutic potential of NMN to treat metabolic and aging-related diseases, the clinical and toxicological evidence to support its utility is currently insufficient (Poddar et al., 2019). Thus, further research is needed to increase the prospects of developing drugs based on NMN.

## Perspective

Among various NAD+ precursors, NMN and NR seem to increase NAD levels more effectively than NAM in rodents (Rajman et al., 2018). Since NAM acts as a feedback inhibitor to suppress sirtuins and PARPs, the benefits of increase in NAD+ levels may be compromised (Bitterman et al., 2002). Additionally, because of the shorter resident time, high-dose administration, and some side effects of NAM, it was not the preferred choice compared with NMN and NR (Kawamura et al., 2016).

It is difficult to compare NMN with NR, both of which are subjected to first-pass metabolism and the quick conversion to other NAD+ intermediates before uptake *in vivo* (Frederick et al., 2016; Ratajczak et al., 2016). NR, instead of NMN, is unstable and quickly converted into NAM in murine plasma (Ratajczak et al., 2016). Some differences regarding the pharmacological effects of NMN and NR should be noted. As mentioned above, NMN improves cardiac function in a SIRT3-dependent manner in an FRDA cardiomyopathy model (Martin et al., 2017), whereas NR does not (Stram et al., 2017). Exploring the pharmacokinetics of NMN and NR *in vivo* could help determine the optimal concentration of them in different regions and facilitate understanding the mechanisms of their pharmacological actions. Head-to-head study should be performed to compare NMN and NR in the future.

Besides making a choice between NMN and NR, more precise data about the dosage and more detailed mechanism are needed. Some researches attempted to utilize novel methods to achieve integral understanding (Assiri et al., 2019; Kiss et al., 2020; Xie et al., 2020). In order to understand the complex NMN-related network changes, high-throughput methods are indispensable, such as methylation profiling, transcriptome, proteome, and metabolomics. In addition, the multifunctional role of NAD+ indicates that NAD+ might function in various organelles. It would be intriguing to decipher NAD+-related mechanism in subcellular compartment.

Further researches are compelling to reveal links between NAD+-related pathway and other disease-related pathway. More importantly, efficiency and safety should be precisely assessed in NMN clinical application.

## Conclusion

NAD+ metabolism has been proven to be an essential part of biochemical reaction that acts as a link between various physiologic processes. During aging, weakened NAD+ biosynthesis and accelerated NAD+ consumption lead to dysfunction in multiple tissues. Depressed NAD+ levels disturb many biochemical processes, for instance, abnormal deacetylation activity of sirtuins. Downstream alterations of abnormal sirtuin activity include transcription pattern, mitochondrial permeability, mtROS production, and oxidative stress response. As an intermediate in NAD+ biosynthesis, NMN is a promising agent to reinforce NAD+ metabolism and alleviate age-related pathologic processes *in vivo*, which has promoted NMN to stage of clinical trial. More details of NAD+ metabolism pathway and applications of NMN are fascinating for further researches.

## Author Contributions

WH wrote the initial manuscript. MH and ZZ created the figures. FM contributed writing material and new ideas. XW revised the manuscript and approved the final version. All authors read and approved the final manuscript.

## Conflict of Interest

The authors declare that the research was conducted in the absence of any commercial or financial relationships that could be construed as a potential conflict of interest.
